# Mechanisms for pattern specificity of deep-brain stimulation in Parkinson’s disease

**DOI:** 10.1371/journal.pone.0182884

**Published:** 2017-08-16

**Authors:** Osvaldo Matías Velarde, Germán Mato, Damián Dellavale

**Affiliations:** Centro Atómico Bariloche and Instituto Balseiro, Consejo Nacional de Investigaciones Científicas y Técnicas (CONICET), Comisión Nacional de Energía Atómica (CNEA), 8400 San Carlos de Bariloche, Río Negro, Argentina; Centre national de la recherche scientifique, FRANCE

## Abstract

Deep brain stimulation (DBS) has become a widely used technique for treating advanced stages of neurological and psychiatric illness. In the case of motor disorders related to basal ganglia (BG) dysfunction, several mechanisms of action for the DBS therapy have been identified which might be involved simultaneously or in sequence. However, the identification of a common key mechanism underlying the clinical relevant DBS configurations has remained elusive due to the inherent complexity related to the interaction between the electrical stimulation and the neural tissue, and the intricate circuital structure of the BG-thalamocortical network. In this work, it is shown that the clinically relevant range for both, the frequency and intensity of the electrical stimulation pattern, is an emergent property of the BG anatomy at the system-level that can be addressed using mean-field descriptive models of the BG network. Moreover, it is shown that the activity resetting mechanism elicited by electrical stimulation provides a natural explanation to the ineffectiveness of irregular (i.e., aperiodic) stimulation patterns, which has been commonly observed in previously reported pathophysiology models of Parkinson’s disease. Using analytical and numerical techniques, these results have been reproduced in both cases: 1) a reduced mean-field model that can be thought as an elementary building block capable to capture the underlying fundamentals of the relevant loops constituting the BG-thalamocortical network, and 2) a detailed model constituted by the direct and hyperdirect loops including one-dimensional spatial structure of the BG nuclei. We found that the optimal ranges for the essential parameters of the stimulation patterns can be understood without taking into account biophysical details of the relevant structures.

## Introduction

Parkinson’s disease (PD) is an chronic progressive neurodegenerative disorder characterized by both motor and non-motor system manifestations. Its pathological definition is loss or degeneration of the dopaminergic neurons in the substantia nigra. This preferential loss of dopamine producing neurons results in marked impairment of motor control. Pharmacological approaches to PD revolve around the imbalances produced by dopamine depletion. However, no definitive disease-modifying therapy to slow or stop the disease exists [1].

High frequency deep brain stimulation (DBS) is a widely used technique for treating motor disorders such as Parkinson’s disease, essential tremor and generalized dystonia [2–9]. In PD, the DBS is considered when drug based therapies such as the administration of levodopa are no longer providing adequate control of symptoms or generate significant side effects such as dyskinesias [10–12]. The open-loop DBS paradigm for movement disorders consists of implanting electrodes (either uni- or bi-laterally) in a given structure, such as the ventrolateral thalamus, the subthalamic nucleus, or the globus pallidus internal section, and continuously applying short duration stimulus pulses at a high frequency (specific frequency range i.e. > 100 Hz). The location of the electrodes depends on the specific pathology [13]. The stimulus has several parameters (polarity, pulse rate, amplitude and width of each pulse) that are adjusted by trained clinicians in order to reduce clinical symptoms while minimizing side effects.

DBS applied to the subthalamic nucleus is effective at improving motor symptoms of PD. Although the mechanisms underlying the DBS technique are mostly unclear [14, 15], it is often assumed that the main DBS mechanism in connection with symptoms improvement in PD is the suppression of *β*-band oscillations (13-35 Hz in humans) [16]. This is based on the reduction of *β*-oscillations in patients treated with levodopa and the correlation between this reduction and clinical improvement [17–19]. On the other hand, in the off-medication state there is less evidence about the correlation between *β*-band oscillations and clinical symptoms [16]. However, the functional significance of excessive *β*-oscillations in terms of a mechanistic causal connection with the symptoms of PD has yet to be demonstrated.

One of the most striking features of DBS therapy to PD is that the stimulation pattern has to be applied with a very specific set of parameters in order to be effective. Thus, the highest threshold for side effects and the lowest threshold for symptom improvement is typically achieved using stimulation frequencies higher than 100 Hz, pulses of short duration (60-100 *μ*s) and amplitudes between 1 to 3 mA [3, 20–24]. In most cases, other parameter configurations have no effect or aggravate the symptoms [2, 3, 25, 26]. The clinical relevance of the high frequency stimulation (HFS), above 100 Hz, seems to be specially puzzling given that the alleviation of the PD motor symptoms is concurrent with the reduction of exaggerated oscillations in the *β*-band, whose frequency is at least three times lower.

In order to understand this behavior several hypothesis about mechanism of action of DBS have been proposed. For instance, in [27] it is analyzed the blocking of axonal transmission generated by the HFS. In [28] the activation of a resonant cortical circuit caused by antidromic spikes is studied. In this case, the efficacy of the mechanism depends on the resonant frequency of the cortical circuit. Similarly, in [29] it is found that driving the cortex at *γ* frequency (60-90 Hz) can lead to an improvement in motor performance.

Other experimental and detailed modeling studies emphasize the regularity of activity patterns produced by the open-loop DBS paradigm [26, 30–34]. These results indicate that above a critical frequency therapeutic HFS changes the intrinsic bursting activity of the parkinsonian target nuclei, to a high-frequency regular pattern of discharge time-locked to the stimulation. Thus, HFS regularizes neural firing patterns across the cortico-basal-ganglia-thalamo-cortical circuit overriding the pathological oscillations in the *β*-band and the resulting informational lesion prevents the pathological activity from being transmitted within the basal ganglia [31, 34]. In this regard, the results of [33] support the idea that HFS works by regularizing basal ganglia activity and thus lowering the entropic noise floor, enabling more information-rich signal propagation and thereby improving thalamic relay fidelity of motor commands [33].

The main goal of this work is to prove that the main qualitative features of the optimal stimulation parameters can be predicted using a generic system that displays oscillations in the *β*-band. Using this simple system, we show that there is a well defined range for the optimal stimulation frequency and that stimulation becomes less effective as it becomes less periodic. We prove this in a simple model with two interconnected neuronal populations, one excitatory and one inhibitory, which is capable of generating oscillations in the *β*-band. These results are also confirmed in a more detailed model, where the oscillations arise from the recurrent coupling between the cortex, the basal ganglia and the thalamus. The detailed model proposed here is an extension of the Leblois scheme [35], which was originally designed for two motor programs, to the possibility of evoking a continuum of motor programs. In both cases we found that the main relevant mechanism is the resetting of the oscillatory activity by the periodic stimulation pattern. We also analyze additional possibilities, such as zero-pole cancellation and suppression of the activity in some of the nuclei. We found that zero-pole cancellation does not suppress the neural activity but it is not successful for reducing oscillations in the *β*-band. In the “Discussion” section we also compare the results of our models with previous explanations for selectivity of the DBS patterns.

## Materials and methods

As described above, motor symptoms of Parkinson’s disease arise from basal ganglia dysfunction. The basal ganglia are a highly organized network constituted by four main subnuclei: striatum, globus pallidus (internal and external segments), subthalamic nucleus, and substantia nigra (compact and reticular). The BG network is critical for motor planning and action selection, but is also involved in associative learning, working memory and emotion [36]. The motor circuit of the BG has several loops, where cortical and subcortical projections interact with internal reentry loops forming a complex network which is thought to be intended for selecting and inhibiting simultaneously occurring events and signals [37]. For this work, we present two models with firing rate dynamics under DBS stimulation. The first is a reduced mean-field model, while the second is an extension of the Leblois model [35] capable of evoking a continuous of motor programs.

### Reduced model

**Fig 1:**
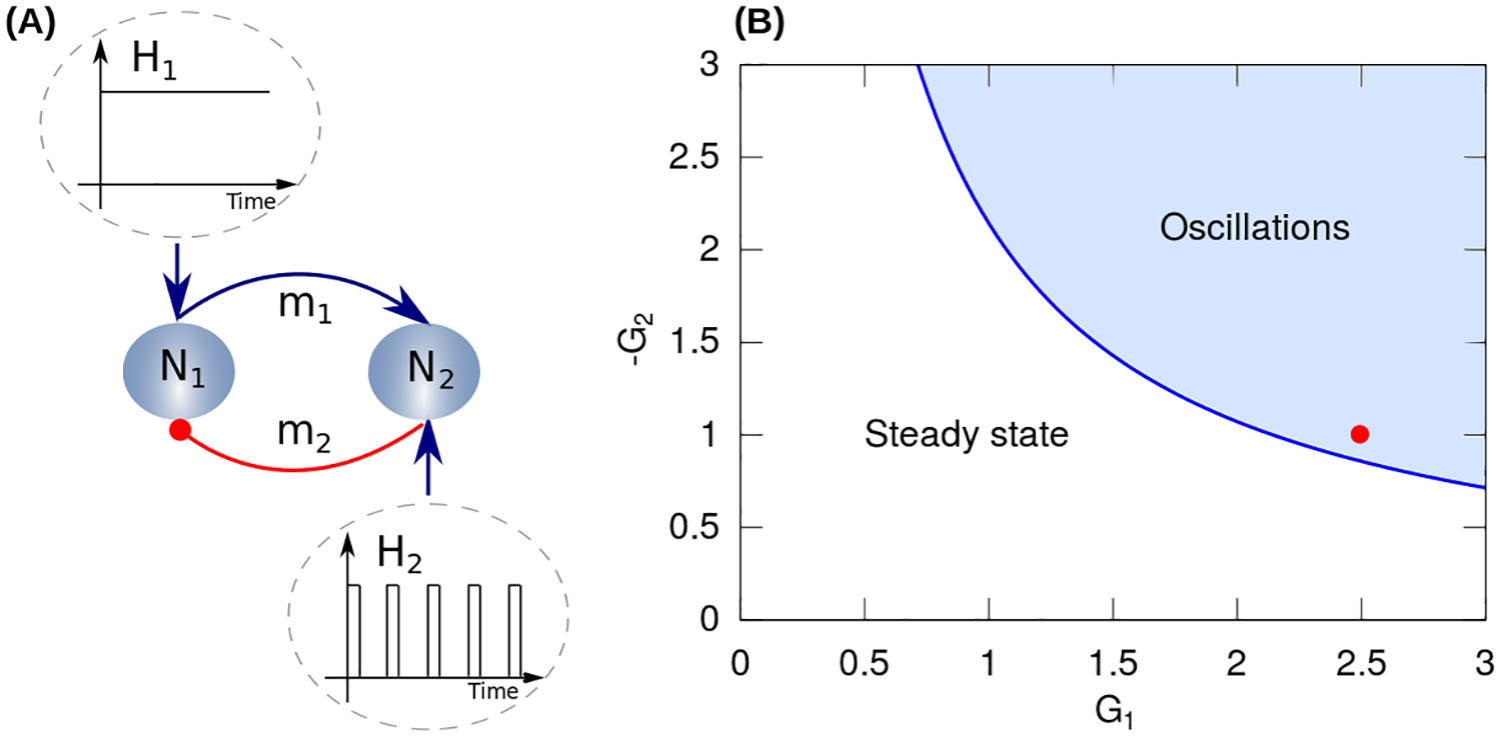
Reduced model. (A) The architecture of the reduced model consists of a single excitatory *N*_1_ and a single inhibitory *N*_2_ neural population that are reciprocally connected, constituting a minimal version of a system capable of generating oscillations. The excitatory and inhibitory projections are indicated by the blue arrow and the red circle, respectively. The neuronal population *N*_1_ receives a constant synaptic input *H*_1_. On the other hand, the neuronal population *N*_2_ receives an external current *H*_2_ constituting the electrical stimulation pattern (DBS signal). (B) Phase diagram of the intrinsic dynamics of the reduced model. The parameters *G*_1_ and *G*_2_ correspond to the synaptic efficacies of the efferent projections of the neural populations *N*_1_ and *N*_2_, respectively. The thick blue line corresponds to the Hopf bifurcation above which the system shows an oscillatory activity (light blue area). The red dot indicates the values of the synaptic efficacies used in the simulations (see Table 1).

In this section we define an analytically tractable model following the framework of minimalistic approach to modeling in order to capture the essential features of the loops present in the BG network. The reduced model consists of a single excitatory and a single inhibitory neural population that are reciprocally connected (see Fig 1A). This representation follows the model introduced in [38], it is a minimal version of a system capable of generating oscillations [38, 39] and represents a prevalent architecture throughout the BG network. The dynamics of the two populations can be written as,

$$\begin{array}{ccc} \tau_{1}{\dot{m}}_{1} & = & -m_{1}+{\left[G_{2}m_{2}\left(t-\Delta_{2}\right)+H_{1}-T_{1}\right]}_{+} \end{array}$$ (1)

$$\begin{array}{ccc} \tau_{2}{\dot{m}}_{2} & = & -m_{2}+{\left[G_{1}m_{1}\left(t-\Delta_{1}\right)+H_{2}-T_{2}\right]}_{+} \end{array}$$ (2)

where *m*_*i*_ and *τ*_*i*_ represent the synaptic output of the population *i* and the time constant of the synapses, respectively. The output of this zero-dimensional representation is constituted by the synaptic currents *I*_1_ = *G*_2_*m*_2_(*t* − Δ_2_) + *H*_1_ and *I*_2_ = *G*_1_*m*_1_(*t* − Δ_1_) + *H*_2_, where *G*_*i*_ indicates the synaptic efficacy of the interactions, *H*_*i*_ is a external input and Δ_*i*_ are delays in the transmission of the interaction.

Regarding the instantaneous activity *A*_*i*_ = *S*_*i*_(*I*_*i*_) of both neural populations, in Eqs 1 and 2 we consider threshold linear transfer functions *S*_*i*_(*I*_*i*_): If *I*_*i*_ > *T*_*i*_, [*I*_*i*_ − *T*_*i*_]_+_ = *I*_*i*_ − *T*_*i*_; else [*I*_*i*_ − *T*_*i*_]_+_ = 0, where *T*_*i*_ is the threshold of population *i* (a further description of the neuron dynamics is given in section “Single Neuron Dynamics” in connection with the detailed model). Besides, one population receives a constant external input *H*_1_, while the other is stimulated by a pulse train *H*_2_ = *H*^*DBS*^.

This model can be solved analytically in the linear state (*I*_*i*_ > *T*_*i*_). Hence, to investigate the stability of the linear steady state, we obtain the equations of the dynamics linearized around that state, i.e. Eqs 1 and 2 for *I*_*i*_ > *T*_*i*_, and analyze the characteristic transcendental equation for the eigenvalues *s*,

$$\begin{array}{c} p\left(s\right)=\left(1+s\tau \right)\left(1+s\mu \tau \right)-Ge^{-s\Delta }=0 \end{array}$$ (3)

where *τ* = *τ*_1_, *τ*_2_ = *μτ*, Δ = Δ_1_ + Δ_2_, *G* = *G*_1_*G*_2_.

The steady state is stable provided that *Re*(*s*) < 0 for all the solutions. It is unstable if at least one solution with *Re*(*s*) > 0 exists. If, for this solution, *Im*(*s*) = 0, the system undergoes a non oscillatory instability. If *Im*(*s*) ≠ 0, the instability is a Hopf bifurcation at a frequency *ω* = *Im*(*s*). Thus, the condition at the onset of the oscillatory instability is given by *p*(*iω*) = 0, or equivalently,

$$\begin{array}{ccc} \tan(\pi -\omega \Delta ) & = & \frac{\omega \tau (1+\mu )}{1-\mu {\left(\omega \tau \right)}^{2}}, \end{array}$$ (4)

$$\begin{array}{ccc} {\left|G\right|}^{2} & = & 1+\left(1+\mu^{2}\right){\left(\omega \tau \right)}^{2}+\mu^{2}{\left(\omega \tau \right)}^{4} \end{array}$$ (5)

Fig 1B shows the phase diagram corresponding to the synaptic efficacies (*G*_1_ and *G*_2_) of the reduced model.

The aim of the reduced model is to analyze the effect of the stimulation’s parameters on the pathological oscillations in the *β*-band (i.e., parkinsonian state). Accordingly, synaptic efficacies *G*_*i*_ were imposed so that the system was in the oscillatory state (red dot in Fig 1B, corresponding to the parameter values reported in Table 1). Besides, by using the time constants reported in [35], the resulting oscillatory activity at 13 Hz belongs to the *β*-band corresponding to parkinsonian humans (13-35 Hz) and nonhuman primate models of PD (8-20 Hz). All the parameters for the reduced model are summarized in Table 1.

**Table 1:** Parameters for the reduced model corresponding to a state of stable oscillatory activity in the *β*-band.

Parameter	Value
*G*_1_	2.5
*G*_2_	-1.0
*T*_1_	0.1
*T*_2_	-0.1
*H*_1_	0.8
*H*_2_	0
Δ_1_	5 ms
Δ_2_	15 ms
*τ*	20 ms
*μ*	0.25

### Network model

In order to examine in a more detailed network the essential results obtained with the reduced model, we use an extended version of the pathophysiology model of PD previously reported in [35]. The model of Leblois et al. [35] was developed as an alternative to the classical model of motor symptoms of PD [40].

The classical model is based on the segregation between the direct and the indirect pathways going from the striatum to BG output structures. This model predicts successfully that inactivation of internal globus pallidus or subthalamic nucleus palliates PD symptoms [41]. However, subsequent experimental findings suggest that this model is incomplete. For instance, the classical connectivity model dictates that external globus pallidus (GPe) ablation would lead to subthalamic nucleus disinhibition, and thus parkinsonian symptoms, which is not observed in the experiments [35].

The limitations of the classical model motivate the development of alternative approaches to address the role the BG play in movement and movement disorders. Thus, the model of Leblois et al. [35] propose the competition between the direct and the hyperdirect loops as a mechanism of the BG-cortex system to perform motor program selection. Under the assumption that dopamine potentiates corticostriatal synaptic transmission, [35] found that the imbalance between the feedback in the direct and hyperdirect loops when dopamine is depleted leads to a loss of action selection ability. Besides, high depletion can lead to synchronous oscillations. As a consequence, this model predicts that the loss of selection ability occurs before the appearance of oscillations, suggesting that Parkinson’s disease motor impairments are not directly related to abnormal oscillatory activity.

In this section we describe our detailed model constituted by the direct and the hyperdirect loops in which we extend the pathophysiology model of PD, previously reported in [35], by including spatial structure to the BG nuclei. Thus, the resulting BG-thalamocortical network model is capable to evoke a continuum of motor programs. Besides, the pathological oscillations seen in PD arise as an emergent property of the proposed detailed model for the BG network upon dopamine depletion.

**Fig 2:**
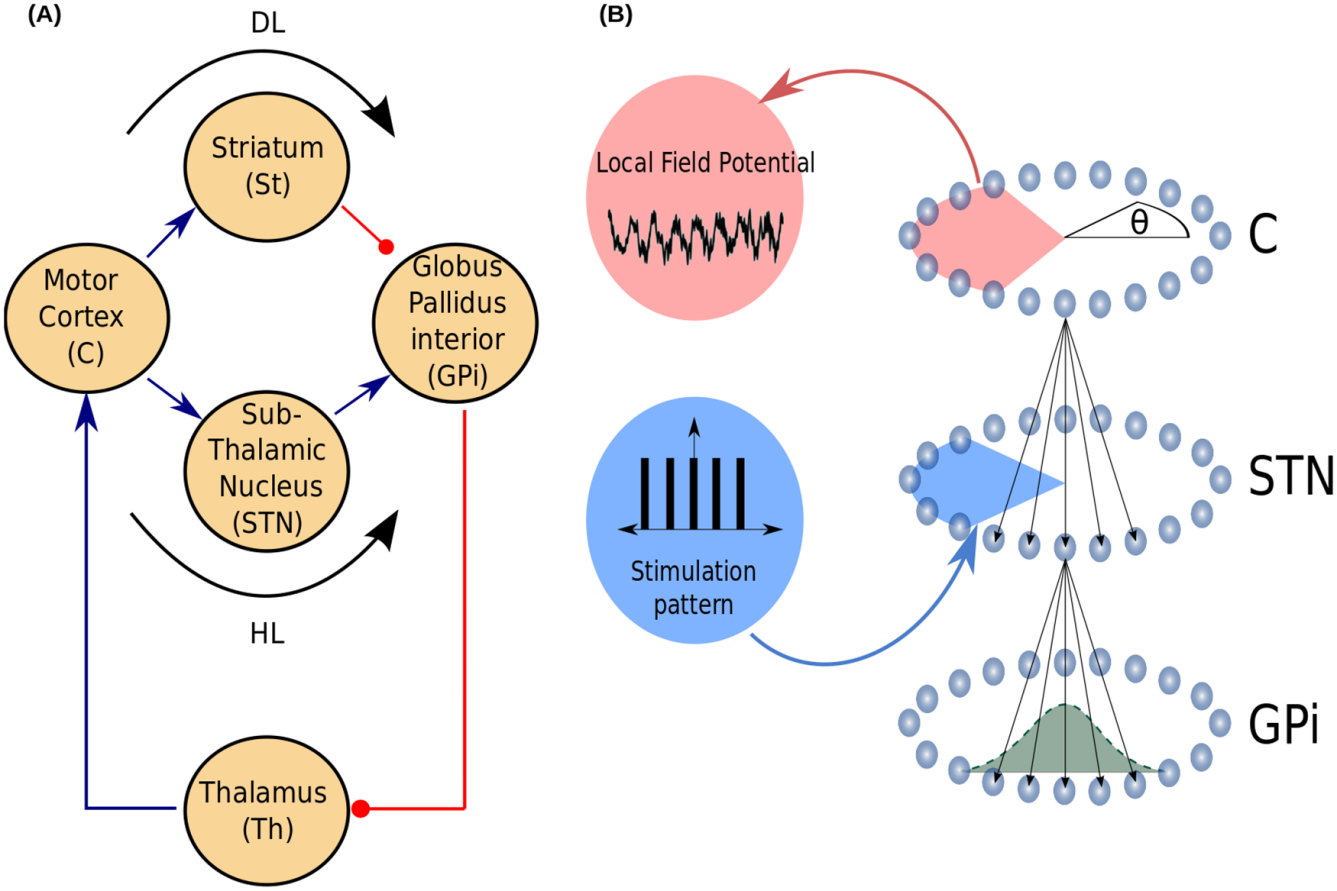
Network model. (A) The architecture of the model is composed of five populations of neurons. Three populations (C, Th, STN) are constituted by excitatory neurons, while the other two (St, GPi) are sets of inhibitory neurons. The excitatory and inhibitory projections are indicated by blue arrows and red circles, respectively. The model includes the hyperdirect (C-STN-GPi) and the direct (C-St-GPi) pathways which operate as competing feedback loops, HL and DL, via the efferent GPi-Th-C projections to close the BG-thalamocortical motor loop. (B) The neurons (light blue spheres) of each population are arranged on a ring, so that the model includes one-dimensional spatial structure of the BG nuclei through the angular coordinate *θ*. The effective volume of the tissue activated (angle highlighted in blue) in the STN and the effective volume of neural tissue contributing to the LFP signal in the C (angle highlighted in red) are defined by the function *F* in Eq 11. The probability of connection between two neurons belonging to two connected BG nuclei (green bell-shaped curve in the GPi), depends on the angular distance between those neurons and it is given by the function *h* in Eq 8. Neurons in the same population are not interconnected.

The network model is composed of five populations of neurons. They represent the motor cortex (C), the ventral anterior and lateral nuclei of the thalamus (Th), the subthalamic nucleus (STN), the striatum (St) and the internal globus pallidus (GPi). The first three populations (C, Th, STN) are constituted by excitatory neurons, while the other two (St, GPi) are sets of inhibitory neurons. This architecture incorporates the direct (DL) and hyperdirect (HL) loops of the BG [35]. The architecture of the network is shown in Fig 2, where we also indicate the components of each loop (see Fig 2A):

1. Positive feedback loop or direct loop (DL): C → St → GPi → Th → C. In this case, cortical neurons excite the thalamus via the St and GPi.
2. Negative feedback loop or hyperdirect loop (HL): C → STN → GPi → Th → C. In this case, the thalamus is inhibited by the motor cortex via the STN and the GPi.

Each population (*α* = Th, C, STN, St, GPi) has *N*_*α*_ neurons. In order to provide the network with a notion of spatial distance, we characterize each neuron *i* of population *α* with an angular coordinate $\theta_{i}^{\alpha }=-\pi +\frac{2\pi }{N_{\alpha }}i\;\;\left(i=1,2,...,N_{\alpha }\right)$. To eliminate edge effects from the computation we use periodic boundary conditions (see Fig 2B).

#### Single neuron dynamics

The single neuron dynamics are characterized by a rate model [38]. A neuron *i* is characterized by the instantaneous activity *A*_*i*_ = *S*_*i*_(*I*_*i*_), where *I*_*i*_ is the total synaptic input to the neuron *i* and *S*_*i*_(*I*_*i*_) is the nonlinear input-output transfer function. We consider threshold linear transfer functions, *S*_*i*_ = [*I*_*i*_ − *T*_*i*_]_+_, where *T*_*i*_ is the threshold of neuron *i*. We assume all the neurons in each population have the same threshold [35].

The synaptic interaction from neuron *i* to neuron *j* is denoted by the variable *m*_*ij*_, which is a low-pass filtered version of its instantaneous activity *A*_*i*_ [42] and is described as follows,

$$\begin{array}{c} \tau_{ij}{\dot{m}}_{ij}=-m_{ij}+A_{i}, \end{array}$$ (6)

where *τ*_*ij*_ is the time constant of the synapses and the over-dot indicates derivative with respect to time.

The total synaptic input *I*_*j*_ received by neuron *j* is given by

$$\begin{array}{c} I_{j}=\sum_{i\to j}G_{ij}m_{ij}\left(t-\Delta_{ij}\right)+H_{j}, \end{array}$$ (7)

where Δ_*ij*_ and *G*_*ij*_ denote the synaptic delay and synaptic efficacy of the interaction *i* → *j*, respectively. Each neuron *j* receives an external input given by *H*_*j*_. We assume that all neurons in the cortex receive a constant input $H_{j}^{C}=0.1$, while neurons in the STN receive the DBS signal, which has spatial structure (see section “Local field potentials and stimulation signals”).

Following [35], we have modeled the dopaminergic depletion by altering the efficacy (*G*_*ij*_) of the C → St interactions and the threshold (*T*_*i*_) of the striatal neurons. For the parkinsonian state, corresponding to strong oscillations in the *β*-band, we took values that represent 90% of depletion of dopamine (see values in Tables 2 and 3). This configuration sets the system in a state of strong oscillations in the *β*-band.

**Table 2:** Values of the coupling parameters for the detailed network model.

	Synaptic efficacy *G*(10^−3^)	Delay Δ (ms)	Synaptic time constant *τ*_*αα*′_ (ms)	K	*σ*(rad)
Th-C	5.44	5	5	229	0.75
C-STN	41.66	5	20	24	0.75
C-St	0.02* (0.8**)	6	5	864	0.75
STN-GPi	67.20	5	5	186	1.57
St-GPi	-1000	10	5	12	0.75
GPi-Th	-1.60	5	5	119	0.75

* Parameter values corresponding to the 90% of depletion of dopamine (parkinsonian state). This configuration sets the system in a state of strong oscillations in the *β*-band.

** Parameter values corresponding to the 100% of dopamine (physiologic state). This configuration sets the system in a non-oscillatory state.

**Table 3:** Threshold values of the semi-linear transfer function for the detailed network model.

Basal Ganglia	Threshold T
Th	-0.185
C	0.11
STN	-0.08
St	-0.005* (-0.02**)
GPi	0.35

* Parameter values corresponding to the 90% of depletion of dopamine (parkinsonian state). This configuration sets the system in a state of strong oscillations in the *β*-band.

** Parameter values corresponding to the 100% of dopamine (physiologic state). This configuration sets the system in a non-oscillatory state.

#### Connectivity

The connections between neurons belonging to two connected BG nuclei were implemented randomly and taking into account the geometry of the network. That is the probability of connection between two neurons depends on the angular distance between them (see Fig 2B). We define a function *h* that indicates the probability of connection between neuron *i* ∈ *α* and neuron *j* ∈ *α*′ (*i* → *j*),

$$\begin{array}{c} h\left(\Delta \theta_{ij}\right)=K_{\alpha ,\alpha^{\prime }}\frac{\exp(\frac{\cos(\Delta \theta_{ij})-1}{\sigma_{\alpha ,\alpha^{\prime }}^{2}})}{\sum_{m\in \alpha }\exp(\frac{\cos(\Delta \theta_{mj})-1}{\sigma_{\alpha ,\alpha^{\prime }}^{2}})}, \end{array}$$ (8)

where *K*_*α*,*α*′_ and *σ*_*α*,*α*′_ are the average connectivity and level of divergence between populations *α* and *α*′, respectively. On the other hand, the model does not include interconnections between neurons in the same population.

#### Spike generation

Spike trains for the neuron *i* were generated according to an inhomogeneous Poisson process [35] with an average rate given by its instantaneous firing rate, $\frac{A_{i}}{\tau_{0}}$, where *A*_*i*_ is the dimensionless instantaneous activity of the neuron *i* and *τ*_0_ defines the temporal scale of the system. That is, the probability that neuron *i* fires a spike at time (*t*, *t* + *dt*) is $A_{i}\frac{dt}{\tau_{0}}$. In this paper, we use *τ*_0_ = 1 ms. In our model, the neuronal refractory period was not taken into account.

#### Local field potentials and stimulation signals

The local field potential (LFP) and electrical stimulation signals are modeled including a spatial modulation given by a function *F*(*θ*_*i*_ − *θ*), where |*θ*_*i*_ − *θ*| is the angular distance between the coordinate of the neuron *i* (*θ*_*i*_) and the location of the electrode (*θ*) [43]. The effective volume of the tissue activated (VTA) and the effective volume of neural tissue contributing to the LFP signal are defined by the function *F*.

In our model, the LFP signal Φ(*θ*^*C*^, *t*) is obtained from the motor cortex nucleus *C* through the following equation

$$\begin{array}{c} \Phi \left(\theta^{C},t\right)=\sum_{i\in C}F\left(\theta_{i}^{C}-\theta^{C}\right)I_{i}\left(t\right) \end{array}$$ (9)

where *θ*^*C*^ and *θ*_*i*_ are the angular coordinates of the electrode and the neuron *i* ∈ *C*, respectively.

On other hand, the stimulation electrode provides an external input $H_{i}^{STN}$ to the neurons of the STN which constitutes the stimulation target,

$$\begin{array}{c} H_{i}^{STN}\left(t\right)=F\left(\theta_{i}^{STN}-\theta^{STN}\right)H^{DBS}\left(t\right). \end{array}$$ (10)

In Eq 10, *H*^*DBS*^ denotes the temporal pattern of the stimulation. In particular, we study regular (i.e., periodic) as well as irregular (i.e., aperiodic) stimulation patterns (see section “Stimulation pattern”).

For both the cases of recording and stimulation, *F*(*θ*_*i*_ − *θ*) is proportional to the periodic version of the Cauchy distribution,

$$\begin{array}{c} F\left(\theta_{i}-\theta \right)=\frac{1}{1-\left(1-\frac{1}{p}\right)(\frac{\cos(\theta_{i}-\theta )-1}{\cos(\theta_{ef}/2)-1})}. \end{array}$$ (11)

where *θ*_*ef*_ represents the VTA or the effective volume of the tissue contributing to the LFP synthesis in the case of the stimulation and the recording electrodes, respectively. If the distance between the neuron *i* and the electrode is less than $\frac{\theta_{ef}}{2}$, the contribution *F* is greater than the fraction threshold *p*.

The Cauchy distribution is consistent with the results reported in [43], that is, the contribution to the LFP of a neuron located far enough from the electrode decays as $\frac{1}{r^{2}}$, where *r* is the distance from the electrode.

#### Stimulation pattern

In the case of regular stimulation patterns we use periodic trains of monopolar pulses, rectangular and triangular in shape, with fundamental frequency *f*_*DBS*_, pulse width *δ* and amplitude $H_{0}^{DBS}$. On the other hand, several classes of irregular stimulation patterns were constructed and denoted by their degree of variability *v*_*f*_. Each irregular DBS train was constructed as a memoryless point processes where the time from one pulse to the next was a random variable 1/*f*. Instantaneous frequencies values *f* were found by drawing a random sample from a Γ distribution [33]. All the Γ distributions used here had a mean value of 〈*f*〉 = 130 Hz. The probability density functions corresponding to the instantaneous frequency were described by

$$\begin{array}{c} \rho \left(f\right)=\frac{\lambda^{k}}{\Gamma (k)}\exp\left(-\lambda f\right)f^{k-1}, \end{array}$$ (12)

where Γ denotes the gamma function, $k=v_{f}^{-2}$ the shape parameter and $\lambda =\frac{k}{\langle f\rangle }$ the scale parameter.

### Simulation procedures

The simulations were performed by integrating the dynamics using a standard first-order Euler algorithm with fixed time step Δ*t* = 0.5 ms. Each simulation was run for 6 s epochs and the first 2.5 s of the simulation time were ignored in order to analyze the steady state. In the case of the detailed model, we took *N*_*α*_ = 2800, ∀*α*. We took values of time constants (*τ*_*α*,*α*′_), delays (Δ), thresholds (*T*) and average connectivity (*K*) as reported in [35], while values of synaptic efficacy (*G*) and level of divergence (*σ*) were obtained by scaling the values reported in [35]. All these parameter values are detailed in Tables 2 and 3. As mentioned in [35], these parameters reproduce the experimental results reported in [44]. In our detailed model, the resulting oscillatory activity at ∼10 Hz closely approximates the *β*-band corresponding to parkinsonian humans (13-35 Hz) and nonhuman primate models of PD (8-20 Hz).

Only two populations receive external input, these are the motor cortex and the STN. We include an external input $H_{i}^{C}=0.1$ representing synaptic entries to the motor cortical neurons *i* = 1,…,*N*_*α*_ coming from other cortical areas and brain regions that are not explicitly incorporated in the model. On the other hand, the external input $H_{i}^{STN}$ represents the stimulation current provided by the DBS electrode on neurons *i* of the STN (Eq 10).

Both, the LFP recording electrode at the motor cortex and the stimulation electrode at the STN, have been positioned at the angular coordinate *θ*^*C*^ = *θ*^*STN*^ = 0 with a fraction threshold of *p* = 0.01, whereas the effective volume *θ*_*ef*_ was obtained from experimental data for volumes of the BG nuclei (S1 Appendix).

### Analysis methods

In order to quantify the neuronal oscillations observed in the simulations, the power spectral density estimator (*PSD*) was computed for the LFP signals using the modified periodogram method [45] with a Gaussian window in time domain. We then compute the *β*-Power as the mean value of the *PSD* around the *β*-band (10-20 Hz), normalized with respect to the *β*-Power obtained in the pathological condition without DBS. In addition, we characterized the activity of the nuclei constituting the BG models by computing the root mean square value $A_{i}^{rms}$ of their corresponding instantaneous activity *A*_*i*_. This quantity incorporates information from all the Fourier modes of the activity, in contrast to the average value, that depends only on the 0-th Fourier mode.

## Results

### Pole-zero cancellation mechanism in the reduced model

We consider the properties of the reduced model in order to assess under which conditions periodic stimulation can lead to the suppression (total or partial) of the pathological oscillations in the *β*-band. We first analyze the situation where the system is in the linear state: *I*_*i*_ > *T*_*i*_ (*i* = 1, 2) by taking the unilateral Laplace Transform on both sides of the differential equations (see “Materials and methods”—Eqs 1 and 2),

$$\begin{array}{c} \tau_{i}s{\hat{m}}_{i}\left(s\right)=-{\hat{m}}_{i}+G_{j}{\hat{m}}_{j}\left(s\right)e^{-s\Delta_{j}}+{\hat{H}}_{i}\left(s\right)-\frac{T_{i}}{s}. \end{array}$$ (13)

where we consider the initial condition *m*_*i*_(*t*) = 0 if *t* ≤ 0 and *i*, *j* = 1, 2, *j* ≠ *i*.

Solving the linear system, we obtain

$$\begin{array}{c} {\hat{m}}_{i}=\frac{G_{j}e^{-s\Delta_{j}}r_{j}+\left(1+\tau_{j}s\right)r_{i}}{p(s)}, \end{array}$$ (14)

where $r_{i}={\hat{H}}_{i}\left(s\right)-\frac{T_{i}}{s}$.

In the context of control theory, the poles of ${\hat{m}}_{i}$ define the dynamics of the system. The poles of the function ${\hat{m}}_{i}$ are the zeros of the transcendental function *p*(*s*) given in Eq 3. In particular, for *p*(*iω*) = 0 there is a pair of imaginary conjugate poles at ±*iω* that generates oscillatory solutions with frequency $\frac{\omega }{2\pi }$. Under this condition, it is possible to tune the stimulation parameters of *H*_2_ to match the zeros of ${\hat{m}}_{i}$ (Eq 14) with its imaginary conjugate poles at ±*iω* so that the oscillation can be canceled. This mechanism is called *pole-zero cancellation*.

For a pulse train *H*_2_ with fundamental frequency *f*_*DBS*_, width pulse *δ* and amplitude $H_{0}^{DBS}$, the following condition is obtained for the pole-zero cancellation,

$$\begin{array}{c} H_{0}^{DBS}\frac{\sin(\omega \delta /2)}{\sin(\omega /2f_{DBS})}e^{i\omega (\frac{1}{f_{DBS}}-\delta )/2}=z\left(H_{1},G_{1},G_{2},\mu ,\tau ,T_{1},T_{2},\Delta_{1},\Delta_{2},\omega \right) \end{array}$$ (15)

where *z* is a complex number that depends only on the system parameters (S2 Appendix).

Eq 15 shows that for each set of DBS parameters (*f*_*DBS*_,*δ*,$H_{0}^{DBS}$), there is a single canceled frequency $\frac{\omega }{2\pi }$. The stimulation frequency *f*_*DBS*_ determines two aspects.

**Fig 3:**
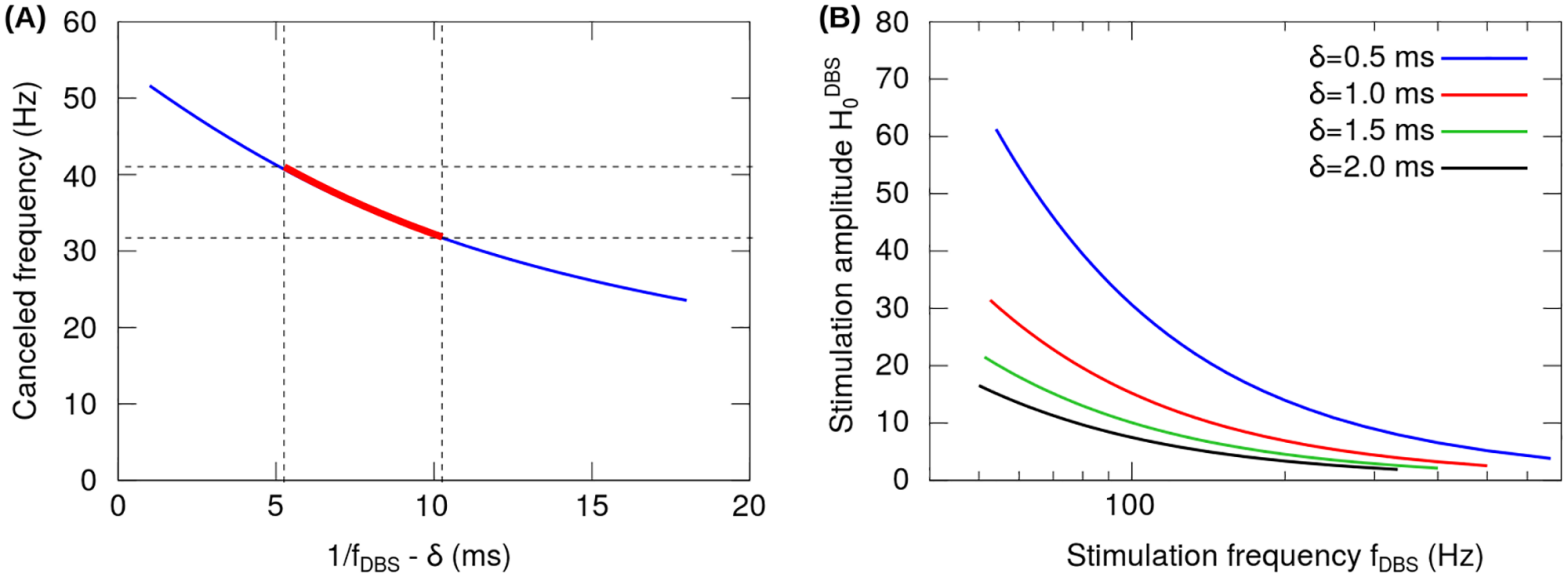
Conditions for pole-zero cancellation. (A) For clinically relevant DBS parameters (*f_DBS_* ≳ 100 Hz, *δ* ∼ 60-100 *μ*s), the pole-zero cancellation produces the total suppression of frequencies above the *β*-band, specifically, in the *γ*-band from 30 to 40 Hz (thick red curve). (B) Stimulation amplitude $H_{0}^{DBS}$ as a function of the stimulation frequency *f*_*DBS*_ for different pulse widths *δ*. This result shows that the required amplitude $H_{0}^{DBS}$ decreases with increasing *f*_*DBS*_ and *δ*, suggesting that the pole-zero cancellation mechanism depends only on the power delivered by the stimulation.

First, by analyzing the conditions for the magnitude and phase emerging from the Eq 15, we obtain that the canceled frequency only depends on the difference $\frac{1}{f_{DBS}}-\delta$. In Fig 3A, for stimulation parameters in the clinically relevant range (*f_DBS_* ≳ 100 Hz, *δ* ∼ 60-100 *μ*s), the pole-zero cancellation mechanism exclusively eliminates oscillations belonging to the *γ*-band. Instead, for *f*_*DBS*_ and *δ* values outside the clinically relevant range ($\frac{1}{f_{DBS}}-\delta >10\text{ ms}$) the pole-zero cancellation mechanism eliminates oscillations belonging to the *β*-band.

Second, for a given canceled frequency, the amplitude $H_{0}^{DBS}$ is univocally determined for the rest of the DBS parameters. Moreover, Fig 3B shows that the amplitude $H_{0}^{DBS}$ decreases with increasing *f*_*DBS*_ and *δ*. This result suggests that the pole-zero cancellation mechanism depends only on the power delivered by the stimulation. In fact, for a constant stimulation power $H_{0}^{DBS}\delta f_{DBS}$, it is obtained that $H_{0}^{DBS}$ results inversely proportional to the *δ*.*f*_*DBS*_ product (Eq 15, data not shown).

### Activity suppression and resetting mechanisms in the reduced model with periodic stimulation pattern

The input synaptic current *I*_1_ to population *N*_1_ was evaluated numerically in response to periodic stimulation patterns *H*_2_ applied on neuronal population *N*_2_ (see Fig 1).

**Fig 4:**
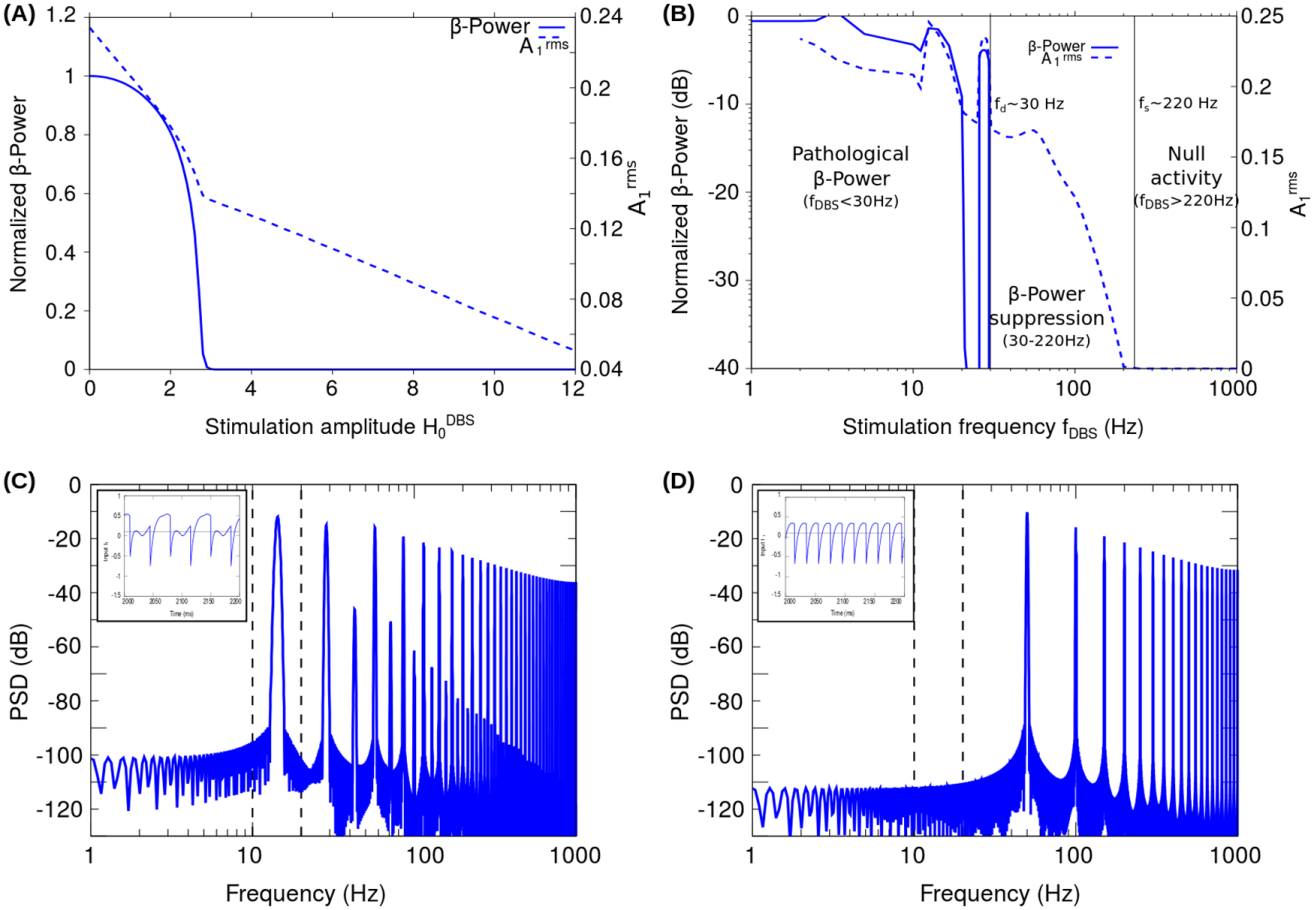
Response of the reduced model in the case of periodic stimulation patterns. The graphs (A-D) show the *β*-Power, root mean square value of the activity and power spectra computed from the synaptic current *I*_1_ corresponding to the neural population *N*_1_, when a periodic stimulation pattern configured with a pulse width of *δ* = 0.5 ms was applied to the population *N*_2_. (A) *β*-Power and the root mean square value of the activity as a function of the stimulation amplitude $H_{0}^{DBS}$ for a constant clinically relevant stimulation frequency of *f*_*DBS*_ = 130 Hz. (B) *β*-Power and the root mean square value of the activity as a function of the stimulation frequency *f*_*DBS*_, keeping the stimulation amplitude unchanged at $H_{0}^{DBS}=10$. The frequency for the period doubling bifurcation *f*_*d*_ ∼30 Hz and the frequency for activity total suppression *f*_*s*_ ∼220 Hz, are indicated with solid vertical lines in order to highlight the transition points between the three states of the system: (1) Null activity (*f*_*DBS*_ > *f*_*s*_), (2) *β*-Power suppression (*f*_*d*_ < *f*_*DBS*_ < *f*_*s*_) and (3) Pathological *β*-Power (*f*_*DBS*_ < *fd*). In the graphs (A) and (B) the *β*-Power has been normalized with respect to that obtained for the system in the oscillatory state without stimulation ($H_{0}^{DBS}=0$). (C) Power spectrum obtained for the stimulation frequency configured at *f*_*DBS*_ = 28 Hz < *f*_*d*_. In this case, the power spectral component in the *β*-band 10-20 Hz (dashed vertical lines), is produced by the period doubling induced by the stimulation frequency. The inset of the graphs show the resulting *I*_1_ signal which has a fundamental period given by the natural frequency of the system $\frac{\omega }{2\pi }=13\text{ Hz}$. (D) Power spectrum obtained for the stimulation frequency configured at *f*_*d*_ < *f*_*DBS*_ = 50 Hz < *f*_*s*_. In this case, the stimulation pattern produces the suppression of the oscillations in the *β*-band without nullifying the activity *A*_1_. Thus, the period of the *I*_1_ signal (1/*f*_*DBS*_) is determined by the stimulation pattern (see the inset) and the resulting power spectral components correspond to the fundamental frequency of the stimulation *f*_*DBS*_ and its harmonics.

In the case of clinically relevant stimulation frequency (*f*_*DBS*_ = 130 Hz) and pulse width (*δ* = 0.5 ms), Fig 4A shows the *β*-Power and the root mean square value of the activity $A_{1}^{rms}$ as functions of the stimulation amplitude $H_{0}^{DBS}$. The PSD estimator and the *β*-Power were calculated from the *I*_1_ signal. The results shown in Fig 4A indicate that, for a clinically relevant frequency *f*_*DBS*_, there does exist a range of DBS amplitudes ($H_{0}^{DBS}∼3-12$) in which it is possible to suppress the pathological oscillations in the *β*-band without nullifying the activity of the neural populations. Unless otherwise specified in the simulations presented here, we take $H_{0}^{DBS}=10$ and *δ* = 0.5 ms.

We also analyzed the effect of the stimulation frequency. Fig 4B shows the dependence of the computed *β*-Power and $A_{1}^{rms}$ with the stimulation frequency *f*_*DBS*_. In Fig 4B, two characteristic frequencies, *f*_*d*_ ∼ 30 Hz and *f*_*s*_ ∼ 220 Hz, are indicated (solid vertical lines) which highlight the transition points between three states of the system: (1) Null activity (*f*_*DBS*_ > 220 Hz), (2) *β*-Power suppression (30 Hz < *f*_*DBS*_ < 220 Hz) and (3) Pathological *β*-Power (*f*_*DBS*_ < 30 Hz).

The frequency for suppression *f*_*s*_ ∼ 220 Hz is the value of the stimulation frequency above which the total suppression of the activity of one of the populations occurs. As a consequence, for *f*_*DBS*_ > *f*_*s*_ the neuronal population *N*_1_ is not active (*A*_1_ = 0), that is, the total synaptic input *I*_1_ is below the activation threshold (*I*_1_ < *T*_1_) in the whole stimulation period ($\frac{1}{f_{DBS}}$). We found that, for a given stimulation amplitude $H_{0}^{DBS}$ and *δ* → 0, the frequency for activity suppression *f*_*s*_ is inversely proportional to the pulse width *δ* (see text below in connection with Eq 16). Therefore, in the case of $H_{0}^{DBS}=10$ and *δ* ∼ 100 *μ*s we obtain *f*_*s*_ ∼ 1.1 kHz. On the other hand, it is important to note that the *β*-Power and $A_{1}^{rms}$ values in Fig 4A and 4B scale with the pulse width *δ* in such a way that the stimulation frequency range for the *β*-Power suppression (30 Hz < *f*_*DBS*_ < 220 Hz) remains unchanged by considering the $H_{0}^{DBS}$ values corresponding to the clinically relevant pulse widths (*δ* ∼ 60-100 *μ*s).

For stimulation frequencies *f*_*DBS*_ below 220 Hz (i.e. 1/*f*_*DBS*_ > 4.5 ms), the activity *A*_1_ becomes zero only in a small fraction of the stimulation period 1/*f*_*DBS*_ related to the response of the system to each current pulse of width *δ* = 0.5 ms (see inset in Fig 4D). Thus, each stimulation pulse produces a resetting of the system activity. As a consequence, the system will be capable to develop oscillations of frequency *f* only if the fundamental period of the stimulation (1/*f*_*DBS*_) is longer than that of the system oscillation 1/*f*. Under this condition, the system has enough time to recover the oscillatory activity between successive resetting pulses.

For the system in the pathological oscillatory state under an external stimulation with *f*_*DBS*_ = 50 Hz, Fig 4B and 4D show that the *β*-Power is negligible compared to that of the system in the oscillatory state without stimulation (*H*_2_ = 0). Accordingly, the state of the system for stimulation frequencies in the range 30 Hz < *f*_*DBS*_ < 220 Hz has been termed as “*β*-Power suppression” (see Fig 4B). It is worth noting that, in the case of *f*_*d*_ > *f*_*DBS*_ > *f*_*s*_, the fundamental frequency of the system response is imposed by the external stimulation (*f*_*DBS*_).

If the stimulation frequency *f*_*DBS*_ is further decreased, a second transition occurs at *f*_*d*_ ∼ 30 Hz, at which a period doubling bifurcation occurs. Specifically, for stimulation frequencies close to *f*_*d*_, the period of the system response is doubled with respect to that of the stimulation, resulting in a increase of the *β*-Power computed from the *I*_1_ signal as shown in Fig 4B. In this connection, Fig 4C shows the power spectra corresponding to *f*_*DBS*_ = 28 Hz in which the spectral peak at 14 Hz is due to the period doubling of the *I*_1_ signal (see inset in Fig 4C). Fig 4B also shows the increase of the *β*-Power produced by tuning the fundamental stimulation frequency in the *β*-band (10 Hz < *f*_*DBS*_ < 20 Hz). For stimulation frequencies *f*_*DBS*_ below 10 Hz, the power of the oscillations in the *β*-band (10-20 Hz) is of the same order of magnitude to that obtained for the system in the oscillatory state without stimulation (*H*_2_ = 0). It was found that for stimulation frequencies *f*_*DBS*_ in almost the whole frequency range below *f*_*d*_ ∼ 30 Hz, no *β*-Power reduction is obtained. Therefore, the state of the system for stimulation frequencies *f*_*DBS*_ below *f*_*d*_ ∼ 30 Hz has been termed as “Pathological *β*-Power” (see Fig 4B).

**Fig 5:**
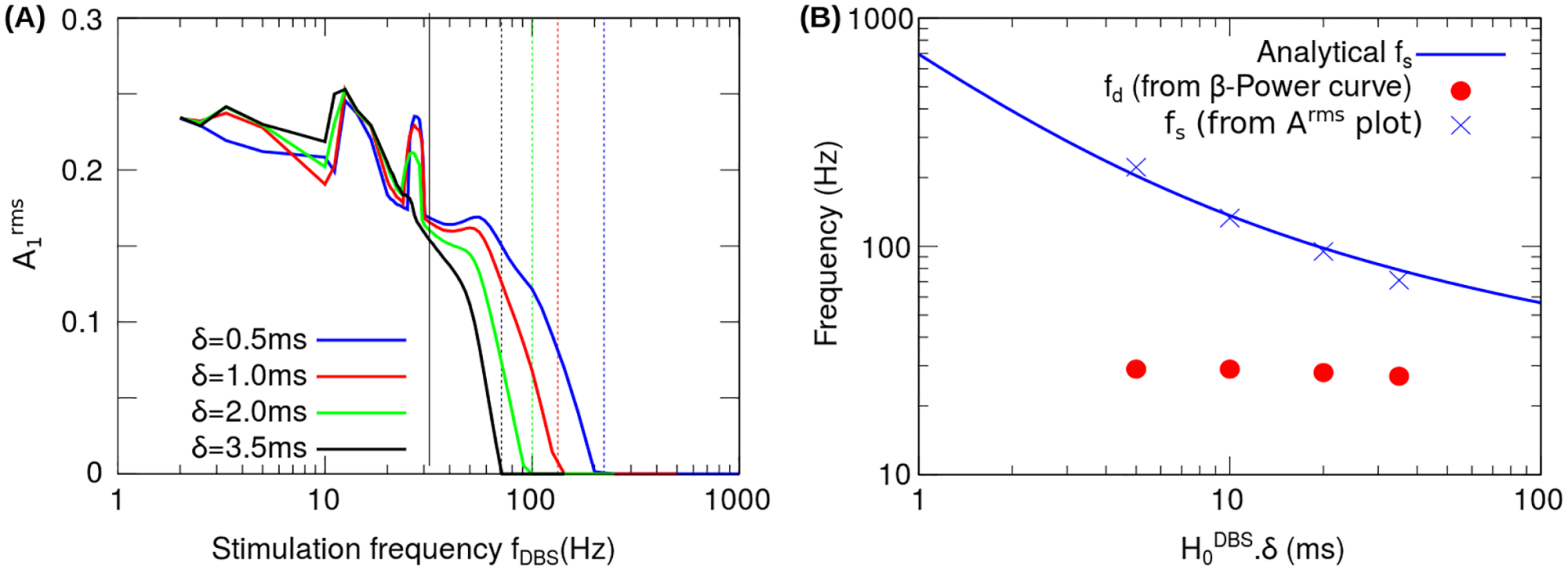
Frequency for activity total suppression (*f*_*s*_) and frequency for period doubling (*f*_*d*_), obtained from the reduced model. (A) Effect on the $A_{1}^{rms}$ of increasing the pulse width *δ* from 0.5 ms to 3.5 ms. The dashed vertical lines indicate the frequency for activity total suppression *f*_*s*_ determined from the $A_{1}^{rms}$ curve. The solid vertical line indicates the frequency for period doubling *f*_*d*_ determined from the *β*-Power curve (Fig 6A). (B) Transition frequencies *f*_*s*_ (blue crosses) and *f*_*d*_ (solid red circles) as functions of the $H_{0}^{DBS}.\delta$ product. The *f*_*s*_ and *f*_*d*_ values were determined from the $A_{1}^{rms}$ plot (Fig 5A) and *β*-Power plot (Fig 6A), respectively. The analytical curve of *f*_*s*_ values (solid blue line) is given by the Eq 16.

In the following, we analyze the effect of the stimulation pulse width *δ* on the transition frequencies *f*_*s*_ (activity suppression) and *f*_*d*_ (period doubling). Fig 5B shows the values of *f*_*s*_ and *f*_*d*_ computed from the *β*-Power and $A_{1}^{rms}$ curves corresponding to four values of *δ* together with the values of *f*_*s*_ obtained analytically from the following equation (S3 Appendix)

$$\begin{array}{c} f_{s}=\frac{1}{\tau_{2}\mathrm{lg}\left(1+H_{0}^{DBS}k\left(e^{\frac{\delta }{\tau_{2}}}-1\right)\right)}∼\frac{1}{H_{0}^{DBS}\delta k}\;\;\left(\delta \to 0\right) \end{array}$$ (16)

where *k* is a combination of the system parameters. The stimulation power required to suppress the neural activity (*A*_1_ = 0) asymptotically approaches to $H_{0}^{DBS}\delta f_{s}∼\frac{1}{k}$ as the pulse width *δ* approaches zero.

**Fig 6:**
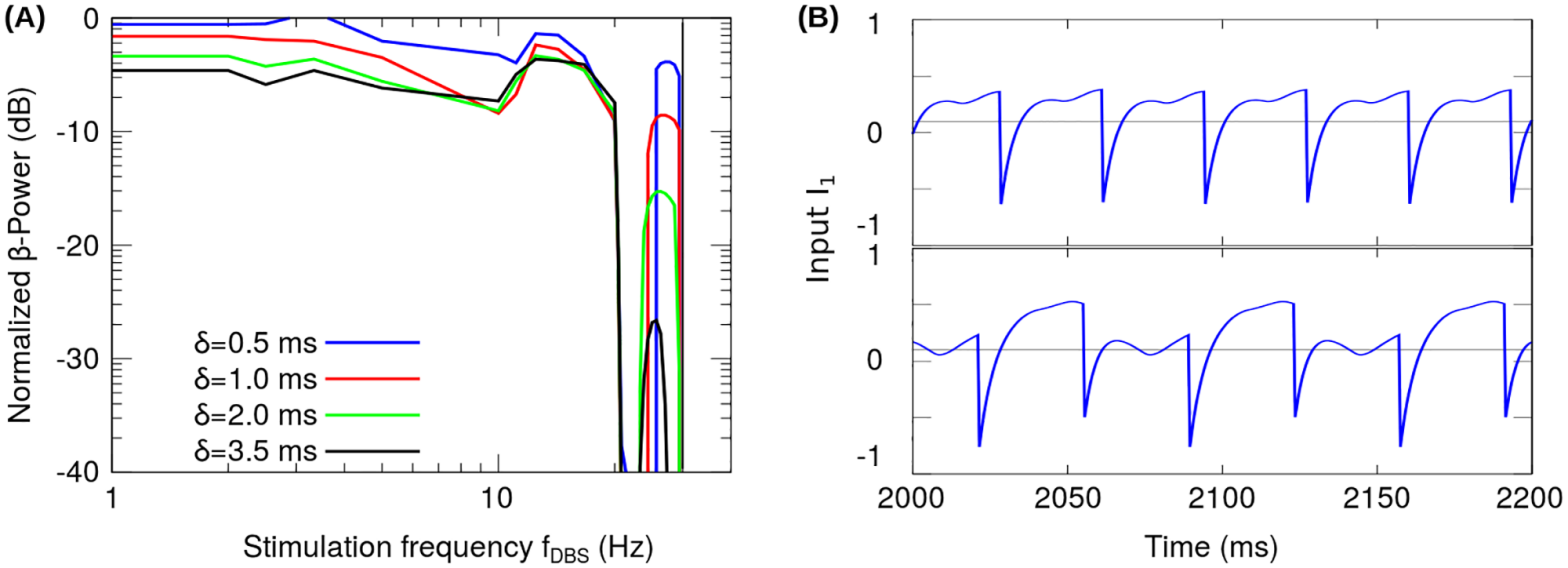
Period doubling transition in the reduced model. (A) *β*-Power as a function of the stimulation frequency (*f*_*DBS*_) using the pulse width *δ* as a parameter. The *β*-Power has been normalized with respect to that obtained for the system in the oscillatory state without stimulation ($H_{0}^{DBS}=0$). The solid vertical line indicates the stimulation frequency at which the period doubling bifurcation occurs (*f*_*d*_). (B) Synaptic current *I*_1_ when *f*_*DBS*_ = 30 Hz + *ϵ* (top), *f*_*DBS*_ = 30 Hz − *ϵ* (bottom), where *ϵ* ∼ 0.6 Hz.

In Fig 5B, the frequency for activity suppression *f*_*s*_ and the frequency for the period doubling *f*_*d*_ are plotted as functions of the product $H_{0}^{DBS}\delta$. The numerical values of *f*_*s*_ shown in Fig 5B (blue crosses) were computed from the $A_{1}^{rms}$ curves corresponding to four values of the pulse width (Fig 5A). Fig 5B shows that these numerical values of *f*_*s*_ are in good agreement with the trend given by the analytical function (Eq 16). On the other hand, the numerical values of *f*_*d*_ were computed from the *β*-Power curves corresponding to four values of the pulse width (Fig 6A). Fig 5A shows that the value of *f*_*d*_ is almost independent of the product $H_{0}^{DBS}\delta$.

**Fig 7:**
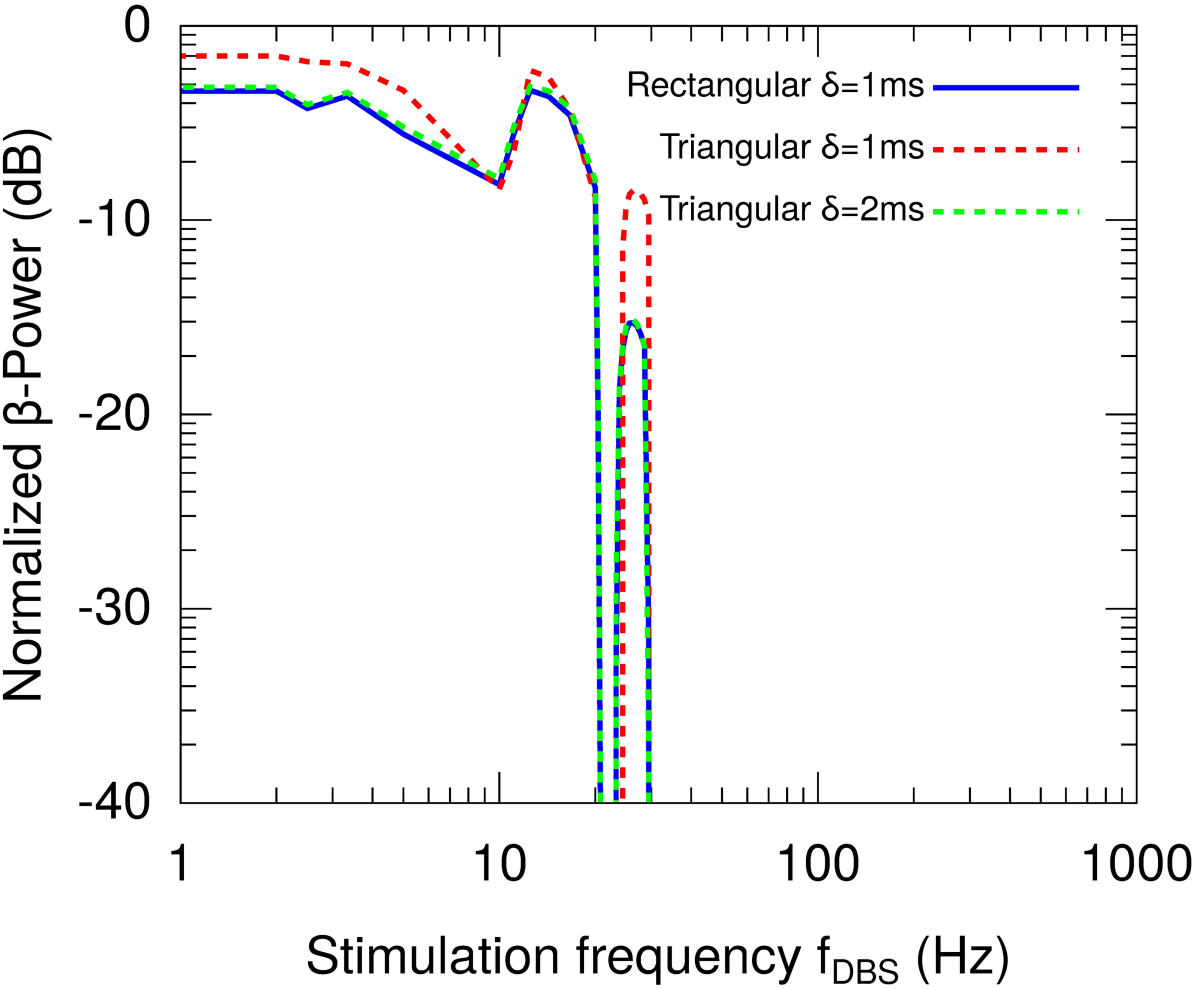
*β*-Power as a function of the stimulation frequency (*f*_*DBS*_) for different pulse shapes of the stimulation pattern. The *β*-Power has been normalized with respect to that obtained for the system in the oscillatory state without stimulation ($H_{0}^{DBS}=0$). This result suggest that the *β*-Power is invariant to the change in the shape of the pulses, and only depends on the area of the pulse constituting the stimulation pattern.

Fourier analysis was applied to analyze the condition for the stimulation frequency at which the period doubling occurs (S4 Appendix). By considering the reduced model in the linear state, it was found for linear case that there is only one stimulation frequency *f*_*d*_ doubling the period of the signal *I*_1_, corresponding to twice the frequency of the system oscillations ($f_{d}=2\left(\frac{\omega }{2\pi }\right)$) and it is independent of the product $H_{0}^{DBS}\delta$. Fig 6B shows the change in *I*_1_ signal due to the period doubling transition (neighborhood of ∼ 30 Hz). It is important to note that in Figs 4B, 6A and 7, a finite range of frequencies can be identified around *f*_*d*_ in which the period doubling occurs. This is due to the nonlinearity of the system equations given by the threshold linear transfer functions in Eqs 1 and 2.

We now want to test that the relevant factor for the results is the power of the stimulation. For this, the system was stimulated with triangular pulse trains in which the pulse amplitude was kept unchanged ($H_{0}^{DBS}=10$) and the pulse width was varied for comparison with the results obtained in the case of a rectangular pulse shape. Fig 7 shows the *β*-Power for rectangular and triangular pulse trains. It is important to note that the power delivered by the triangular pulse *δ* = 2 ms is the same as the rectangular pulse *δ* = 1 ms. Thus, the results shown in Fig 7 suggest that the *β*-Power is invariant to the change in the shape of the pulses, and only depends on the area of the pulse constituting the stimulation pattern.

### Resetting mechanisms in the reduced model with irregular stimulation pattern

**Fig 8:**
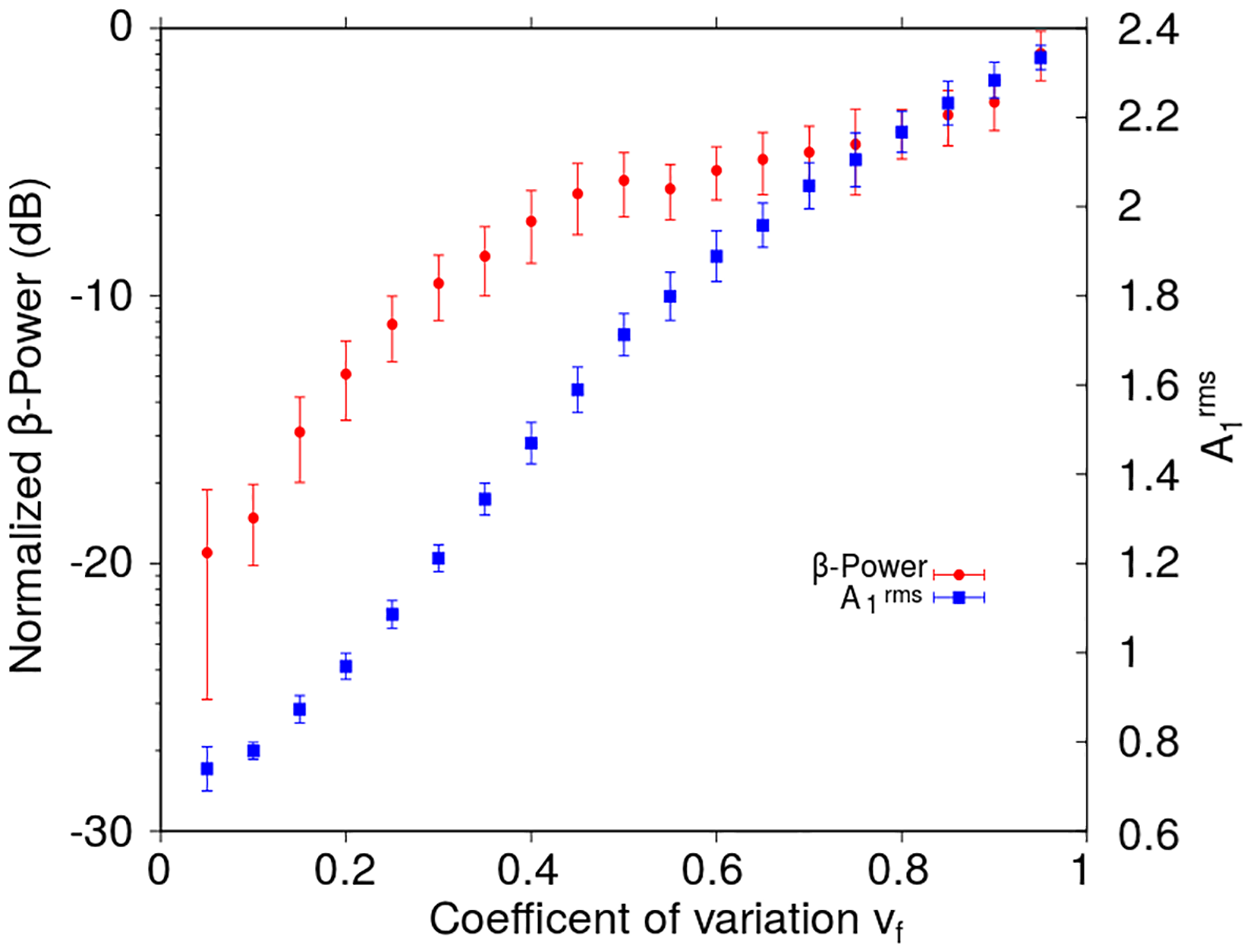
*β*-Power and the root mean square value of the activity $A_{1}^{rms}$ as functions of the coefficient of variation *v*_*f*_, in the case of the reduced model. The *β*-Power and the $A_{1}^{rms}$ were computed from the synaptic current *I*_1_ corresponding to the neural population *N*_1_, for non-periodic stimulation patterns configured with $H_{0}^{DBS}=10$ and *δ* = 0.5 ms applied to the population *N*_2_. The *β*-Power has been normalized with respect to that obtained for the system in the oscillatory state without stimulation ($H_{0}^{DBS}=0$). The non-periodic stimulation patterns were generated using random instantaneous frequencies with a mean value of 〈*f*_*DBS*_〉 ∼ 130 Hz. Each point in the graph corresponds to the mean value and the standard deviation of 10 realizations for each *v*_*f*_ value (see section “Materials and methods”).

In this section we analyze the effect of temporally irregular, i.e. aperiodic, stimulation patterns on the inhibitory-excitatory neural populations constituting the reduced model. Aperiodic trains of rectangular-shaped pulses were generated with mean frequency 〈*f*_*DBS*_〉 ∼ 130 Hz and different coefficients of variability *v*_*f*_. For each *v*_*f*_, 10 pulse trains were built randomly and the system (Fig 1) was stimulated with each of them. Fig 8 shows the mean and standard deviation of the *β*-Power computed from the *I*_1_ signals corresponding to 10 realizations for each *v*_*f*_ value. In all these simulations the amplitude and width of the rectangular stimulating pulses were kept unchanged ($H_{0}^{DBS}=10$, *δ* = 0.5 ms).

In Fig 8, the increase of the *β*-Power as the temporal irregularity of the stimulation pattern (*v*_*f*_) is increased, suggests that a temporally regular stimulation pattern is more effective to reduce the *β*-band oscillations than aperiodic pulse trains (*β*-Power span range of 30 dB in Fig 8). We also observe that the global level of activity, quantified by $A_{1}^{rms}$, is weakly affected by the irregularity of the stimulus ($A_{1}^{rms}$ span range of 6 dB in Fig 8).

The results from the two last sections indicate that the resetting of the amplitude of the oscillations is indeed a plausible mechanism for suppression of excessive oscillations in the *β*-band. Even in this oversimplified framework, it allows us to understand important properties that are found in experimental situations such as the specificity of the stimulation frequency of periodic patterns and the relative ineffectiveness of non-periodic stimulation. The effective frequency range obtained from the reduced model analysis (30 Hz < *f*_*DBS*_ < 220 Hz) is in good agreement with the clinically relevant high-frequency range for DBS in PD (50 Hz < *f*_*DBS*_ < 180 Hz) [13, 26, 46]. In this regard, [3] experimentally showed that the effectiveness in PD tremor suppression of the HFS of the ventral intermediate nucleus began at more than 60Hz, peaked from about 150 to 1000 Hz, and then slowly fell until 5000 Hz. Besides, [47] relates this limit with mechanisms such as blockade of axonal conduction. However, the origin of the upper bound for the clinically relevant stimulation frequency is not fully understood, and could be related, among other factors, with the reduction of the VTA as the pulse width of the stimulation pattern is decreased.

The results obtained with our reduced model indicates that the activity resetting mechanism elicited by an excitatory ($H_{0}^{DBS}>0$) and periodic stimulation pattern support the emergence of a clinically relevant high-frequency range (*f*_*d*_ < *f*_*DBS*_ < *f*_*s*_) for the DBS. Then, the clinically optimal stimulus frequency within this high-frequency range might be determined by more complex mechanism not captured by our reduced model.

### Activity suppression and resetting mechanisms in the network model with regular and irregular stimulation patterns

In order to check some of the predictions obtained with the reduced model (Fig 1), we analyzed the behavior of the rate network model constituted by the direct and hyperdirect loops (Fig 2). For this, we configure the parameters of the network model for 90% of dopamine depletion (see values in Tables 2 and 3) which set the system in the parkinsonian state corresponding to strong oscillations in the *β*-band (10 Hz). Besides, the stimulation pattern was applied to the STN at a constant pulse amplitude $H_{0}^{DBS}=7$. The LFP signal was obtained from synaptic currents of cortical neurons (see Eq 9 in section “Materials and methods”). Since the basal ganglia network model (Fig 2) is constructed from random connections, 10 different realizations were simulated for each stimulation pattern.

**Fig 9:**
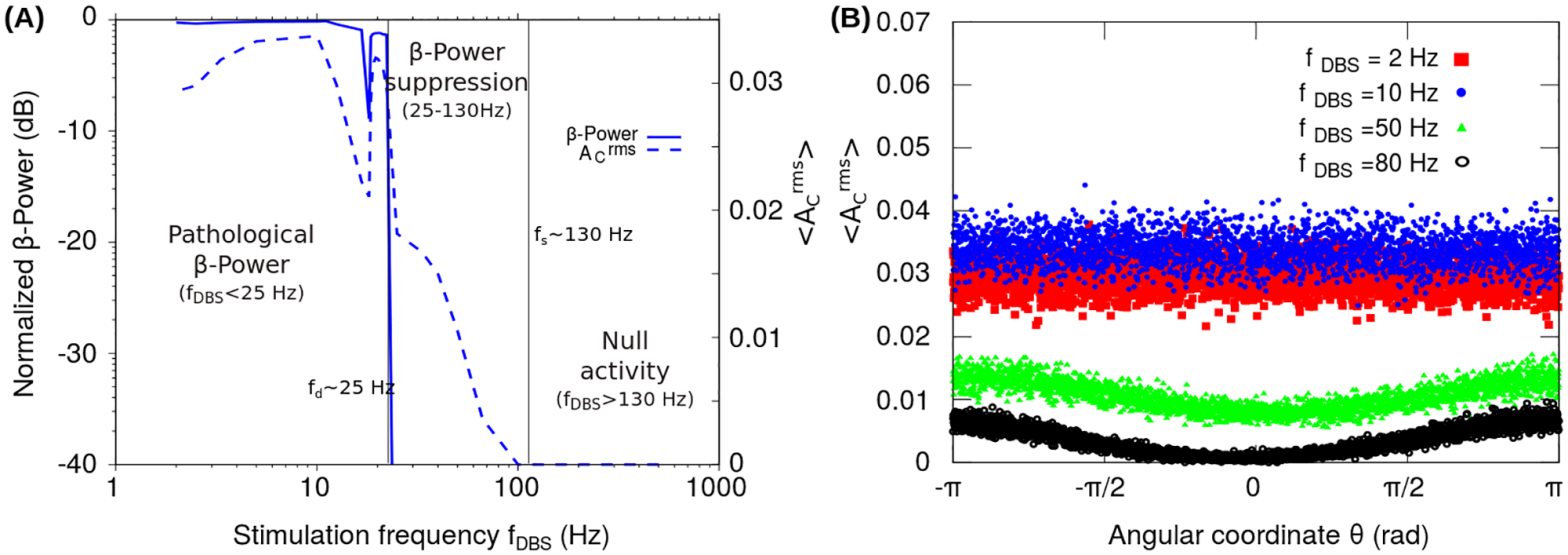
Response of the network model in the case of periodic stimulation patterns. The graphs (A-B) show the *β*-Power and root mean square value of the activity computed from the cortical LFP signal (Eq 9), when a periodic stimulation pattern configured with a pulse width of *δ* = 0.5 ms was applied to the STN nuclei. *β*-Power and the root mean square value of the activity as a function of the stimulation frequency *f*_*DBS*_, keeping the stimulation amplitude unchanged at $H_{0}^{DBS}=7$. The *β*-Power has been normalized with respect to that obtained for the system in the oscillatory state without stimulation ($H_{0}^{DBS}=0$). Besides, the $\langle A_{C}^{rms}\rangle$ correspond to the root mean square value of the activity averaged over all the cortical neurons. The frequency for the period doubling bifurcation *f*_*d*_ ∼ 25 Hz and the frequency for activity total suppression *f*_*s*_ ∼ 130 Hz, are indicated with solid vertical lines in order to highlight the transition points between the three states of the system: (1) Null activity (*f*_*DBS*_ > *f*_*s*_), (2) *β*-Power suppression (*f*_*d*_ < *f*_*DBS*_ < *f*_*s*_) and (3) Pathological *β*-Power (*f*_*DBS*_ < *f*_*d*_).

**Fig 10:**
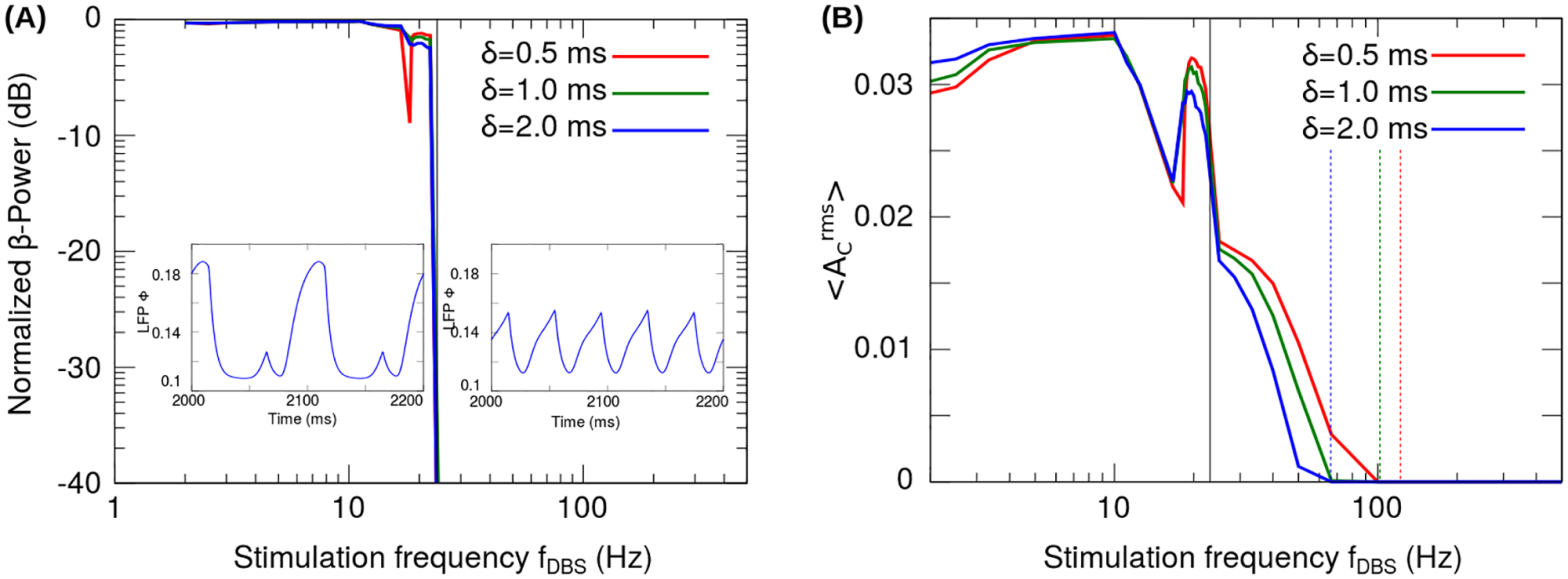
Response of the network model in the case of periodic stimulation patterns. The graphs (A-B) show the *β*-Power and root mean square value of the activity computed from the cortical LFP signal (Eq 9), when a periodic stimulation pattern configured with an amplitude of $H_{0}^{DBS}=7$ was applied to the STN nuclei. (A) *β*-Power as a function of the stimulation frequency *f*_*DBS*_ using the pulse width *δ* as a parameter. The *β*-Power has been normalized with respect to that obtained for the system in the oscillatory state without stimulation ($H_{0}^{DBS}=0$). The solid vertical line indicates the stimulation frequency at which the period doubling bifurcation occurs (*f*_*d*_). (B) Effect on the $\langle A_{C}^{rms}\rangle$ of increasing the pulse width *δ* from 0.5 ms to 2 ms. The dashed vertical lines indicate the frequency for activity total suppression *f*_*s*_ determined from the $\langle A_{C}^{rms}\rangle$ curve. The solid vertical line indicates the frequency for period doubling *f*_*d*_ determined from the *β*-Power curve (graph (A)). The $\langle A_{C}^{rms}\rangle$ correspond to the root mean square value of the activity averaged over all the cortical neurons.

**Fig 11:**
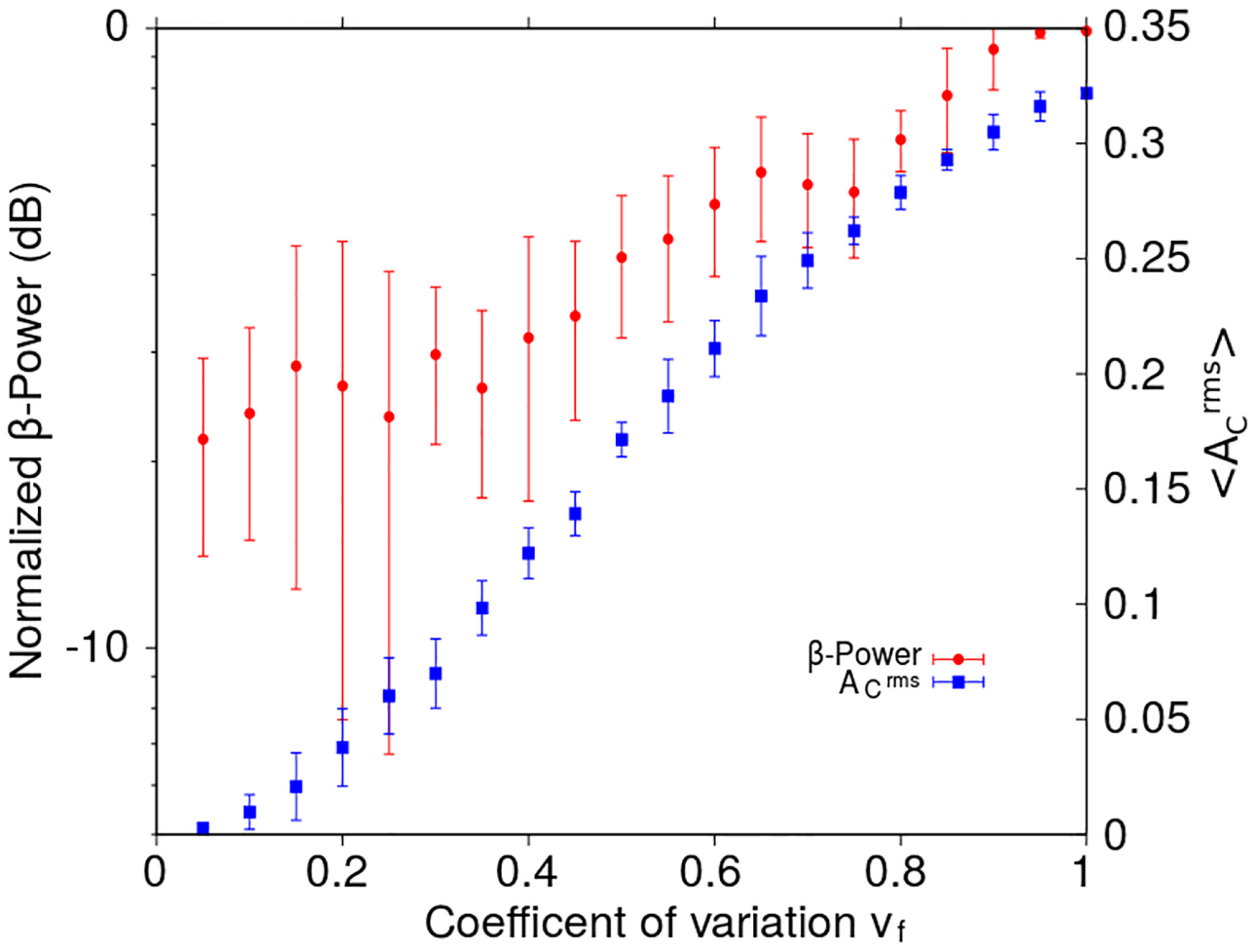
*β*-Power and the root mean square value of the activity as functions of the coefficient of variation *v*_*f*_, in the case of the network model. The *β*-Power and the $\langle A_{C}^{rms}\rangle$ were computed from the cortical LFP signal (Eq 9), when non-periodic stimulation patterns configured with $H_{0}^{DBS}=7$ and *δ* = 0.5 ms were applied to the STN nuclei. The *β*-Power has been normalized with respect to that obtained for the system in the oscillatory state without stimulation ($H_{0}^{DBS}=0$). The $\langle A_{C}^{rms}\rangle$ correspond to the root mean square value of the activity averaged over all the cortical neurons. The non-periodic stimulation patterns were generated using random instantaneous frequencies with a mean value of 〈*f*_*DBS*_〉 = 130 Hz. Each point in the graph correspond to the mean value and the standard deviation of 10 realizations for each *v*_*f*_ value (see section “Materials and methods”).

The results shown in Figs 9, 10 and 11 are equivalent to the ones obtained for the reduced model. Specifically, the three states of the system induced by the frequency of the periodic stimulation are reproduced with the neural network model (Fig 9): Null activity (*f*_*DBS*_ > 130 Hz), *β*-Power suppression (25 Hz < *f*_*DBS*_ < 130 Hz), Pathological *β*-Power (*f*_*DBS*_ < 25 Hz). It is noteworthy that the detailed model reproduces the period doubling bifurcation observed in the response of the reduced model (Figs 4B and 6). In this regard, Figs 9 and 10 show the rise in the activity ($A_{C}^{rms}$) and *β*-Power around *f*_*DBS*_ ∼ 30 Hz corresponding to the period doubling bifurcation occurring in the detailed model. Fig 10 also shows that, similarly to the reduced model, the frequency for period doubling is almost independent of the $H_{0}^{DBS}\delta$ and the frequency for activity suppression decreases as the pulse width of the stimulation pattern is increased.

Moreover, the results shown in the Fig 11 suggest that a temporally regular stimulation pattern is more effective for reducing the *β*-band oscillations than aperiodic pulse trains with *f*_*DBS*_ ∼ 130 Hz, consistently with previously reported numerical simulations and experimental observations in connection with DBS applied on the STN in parkinsonian patients [33].

**Fig 12:**
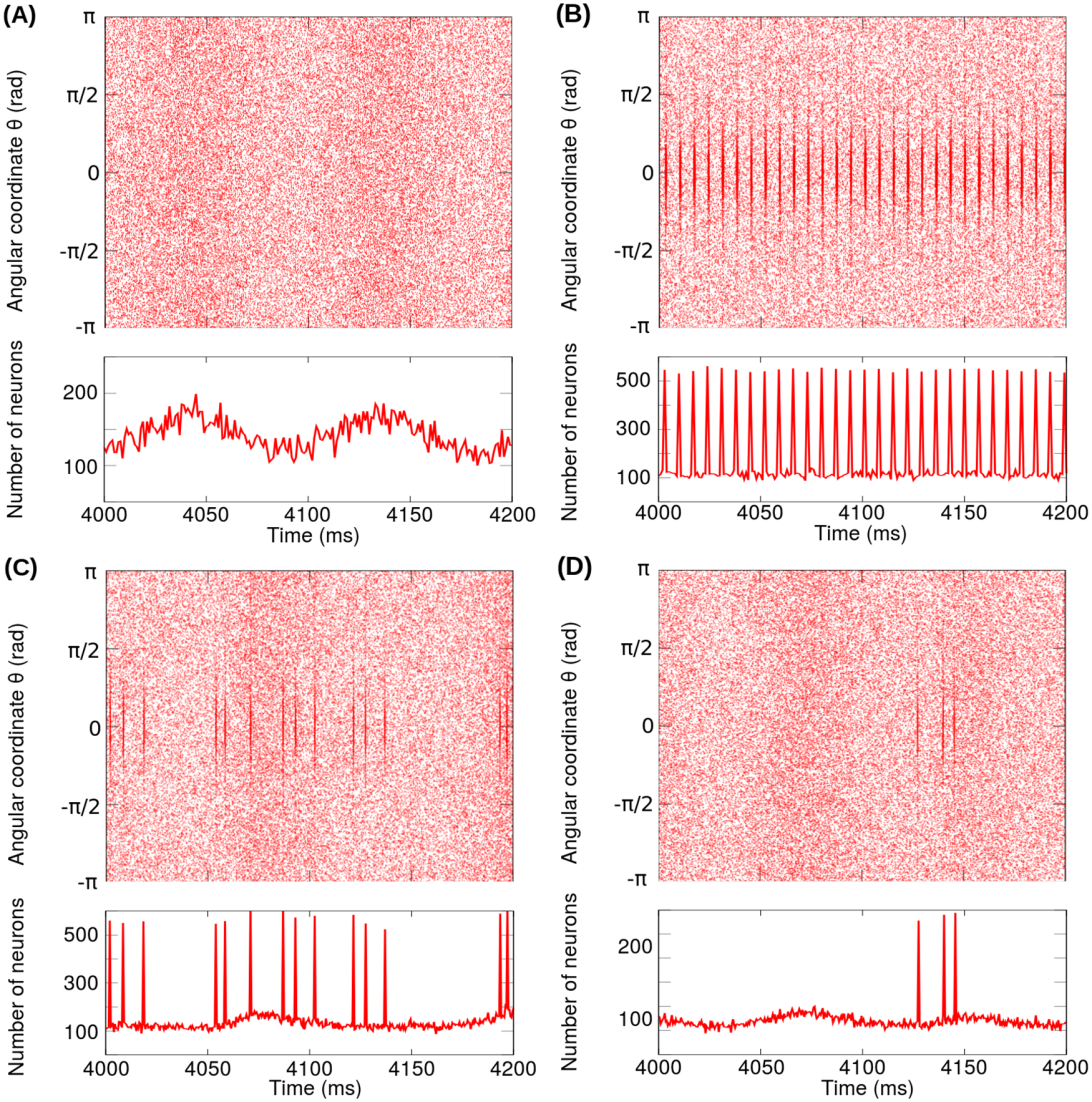
Spike rasters together with the spike histograms for STN neurons corresponding to the network model. (A) Pathological oscillatory state without stimulation ($H_{0}^{DBS}=0$). (B) Pathological oscillatory state under a periodic stimulation pattern with *f*_*DBS*_ = 130 Hz. (C) Pathological oscillatory state under a non-periodic stimulation pattern with 〈*f*_*DBS*_〉 = 130 Hz and *v*_*f*_ = 90%. (D) Pathological oscillatory state under a non-periodic stimulation pattern with 〈*f*_*DBS*_〉 = 130 Hz and *v*_*f*_ = 70%. In the plots (B), (C) and (D), the stimulation patterns were applied on the STN nuclei and configured with $H_{0}^{DBS}=7$ and *δ* = 0.5 ms.

Fig 12A shows the spike rasters together with the spike histograms for STN neurons corresponding to the pathological oscillatory state without stimulation. In Fig 12A, the spike histogram reveals the oscillation in the *β*-band (10 Hz) corresponding to the pathological state of the network model (see Tables 2 and 3). It is worth noting that the application of a periodic stimulation pattern with fundamental frequency *f*_*DBS*_ ∼ 130 Hz on the STN give rise to the suppression of the pathological oscillation (10 Hz) without nullifying the activity of the STN or the motor cortex. In this regard, Fig 12B explicitly shows that the subthalamic neurons belonging to the volume of tissue activated, about 50% of the STN neurons (S1 Appendix), fire synchronously with the periodic stimulation pattern. In the case of non-periodic stimulation patterns, Fig 12C and 12D show that the oscillatory activity in the *β*-band increases and the firing regularity of the STN neurons decreases following the increase of the coefficient of variation *v*_*f*_ (see also Fig 11).

The results of this section are completely compatible with the ones obtained for the reduced model: we have a well defined range of frequencies for the periodic stimulation patterns and less efficacy of the aperiodic stimulation.

## Discussion

Since pioneering works [2, 3] demonstrated the clinical effectiveness of the DBS in the treatment of medically refractory movement disorders, a considerable amount of theoretical and experimental scientific investigations have been devoted to elucidate the mechanism of action of the HFS on the BG-thalamocortical network.

As a result, four general hypotheses have been proposed for the mechanism of action of DBS, which in turn can be further subdivided in several specific mechanisms: 1) Inhibition caused by depolarization-induced blockade and inactivation of voltage-gated currents [48], synaptic inhibition due to the activation of inhibitory GABAergic afferents in the stimulated nucleus [49]. 2) Activation by excitation of efferent pathways [30], antidromic excitation of cortex projections [28, 29]. 3) Inhibition and activation (*antidromic*, *orthodromic*) of target neurons by uncoupling inhibition of soma and dendrites, and activation of axons [32, 50], local functional inhibition by neurotransmitters depletion and excitation of efferent axons by the electrical stimulus [26]. 4) Disruption (*jamming*) of the information flow [26, 31, 34, 51], activity complex locking with the stimulation pattern [33], suppresses low-frequency network oscillations [52], loop-based reinforcement [53], stimulation-induced blockade of axonal conduction [27].

All this evidence suggests that the identified ways of action of DBS might be involved simultaneously or in sequence and that the relevance of each specific mechanism depends on the DBS configuration, that is, the stimulus parameters (e.g., frequency, intensity), the neural tissue composition of the target nuclei and electrode assembly used in the experiments. Many of these experimental studies are specifically related to the emergence of the clinically relevant high-frequency range for electrical stimulation in PD [3, 26–28, 30, 49].

In this work, we have shown that the specific range for both the frequency and the intensity for which the external stimulation is capable to change the intrinsic bursting activity of the target nuclei to a high-frequency regular pattern of discharge time-locked to the stimulation, is an emergent property of the BG-thalamocortical anatomy at the system-level. In particular, the relevant frequency range for the external stimulation predicted by our reduced model is consistent with those obtained heuristically using open-loop DBS paradigms in parkinsonian patients. It is worth noting that these paradigms are based on criteria of symptom improvement [21–24, 26, 54].

We have shown that the emergent property of the BG-thalamocortical network under electrical stimulation can be studied using mean-field descriptive models of the network, which do not account for the detailed description of individual neurons constituting the DBS target nuclei. Moreover, we have shown that the effect of periodic and aperiodic stimulation patterns can also be successfully addressed using models that do not incorporate the fine temporal resolution of neuronal spiking in favor of average neural activity. This approach contrasts with the one used in other works where experimental evidence is in general compared and interpreted through physiologically realistic computational models [33, 47, 52, 53, 55–60] in order to account for the fact that in the common DBS targets (Th, GPi, STN) therapeutic effectiveness occurs only at high stimulation frequencies (*f_DBS_* ≳100 Hz). In these cases, the specific interpretation depends on the details of the computational model. For instance, in [28] the optimal stimulation frequency for DBS is determined by a resonant frequency in a cortical circuit. Instead, in [27] the frequency is determined by the blockade of axonal conduction.

We have analyzed two different levels of modeling. The first one is a generic oscillatory system composed of two populations (one excitatory and one inhibitory), that can oscillate in the *β*-band because of the transmission delays. The second is a more detailed model that incorporates aspects of the architecture of the basal ganglia-thalamocortical loops, specifically, the direct and hyperdirect loops. The fact that the two approaches give rise to the same qualitative conclusion is an indication of the robustness of our results. Let us note that our detailed model is just one of the possible implementations of the relevant loops. One could also have modeled the direct and indirect loops [40, 61] or considered the oscillations as generated by the STN-GPe subsystem. In fact, for the second case, the interaction between STN and GPe can be represented by neuronal populations *N*_1_ and *N*_2_, respectively (Fig 1A). This is relevant since experimental and theoretical evidence have suggested that the STN-GPe loop formed within the indirect pathway is involved in the genesis of tremor-like oscillations [62] and exaggerated oscillations in the *β*-band related to bradykinesia and rigidity [52, 55, 56, 59, 63–68].

Even though our detailed model exhibits an architecture far more complex than that of the reduced model (see Figs 1 and 2), the activity resetting mechanism elicited by a periodic stimulation pattern is present in both computational models (detailed and reduced) suggesting that: 1) the proposed reduced mean-field model is an elementary building block capable to capture the underlying fundamentals of relevant loops constituting the BG network in connection with previously reported pathophysiology models of PD [35, 40, 52, 55, 56, 62] and 2) the resetting of the oscillatory activity elicited by the periodic stimulation pattern is a common key mechanism at the system-level for clinical relevant DBS configurations.

Let us note that in both models (reduced and detailed), the occurrence of oscillations is possible depending on the synaptic gains. In the reduced model, it can be interpreted as the effect of the total gain between populations, while in the detailed model, as a result of competition between the direct and hyperdirect loop. Interestly, even when the reduced model can be thought as simplified representation of the hyperdirect (negative gain) loop, we found that it is also capable to reproduce the dynamics related to the competition between the globally excitatory direct loop and the inhibitory hyperdirect loop. Specifically, both model (reduced and detailed) reproduces the emergence of the resetting of the amplitude of the oscillations as a system-level mechanism that gives rise to: 1) suppression of beta oscillations, 2) period doubling dynamics elicited by an appropriate range of stimulation frequency and 3) the relative ineffectiveness of non-periodic stimulation.

We used STN as brain target for DBS in the network model because it represents the usual clinical configuration. In the case of the reduced model, we show the results corresponding to an excitatory stimulation applied on the neural population *N*_2_. However, it is important to note that the reduced model reproduces the dynamics related to the competition between the globally excitatory direct loop and the inhibitory hyperdirect loop and the emergence of the clinically relevant high-frequency range (*f*_*d*_ < *f*_*DBS*_ < *f*_*s*_) when the excitatory stimulation pattern is applied either on the neuronal population *N*_1_ (data not shown), or in the neuronal population *N*_2_ (Figs 1–8). Interestingly, in both cases the reduced model predicts a progressive suppression of *β*-Power as the DBS amplitude is increased (Fig 4A), which seems to be consistent with experimental evidence (Figure 3 of [54], Figure 8 of [68]).

On the other hand, since the detailed model considers an additional (direct) loop, there are other mechanisms (e.g. the selection of motor programs) that can not be analyzed in the reduced model (one more node should be added). It is important to note that the system presented by Leblois et al. [35] is at an intermediate level between the reduced model (2 x 1 dimensional) and the detailed model (5 x 2800 dimensional). The system presented by Leblois et al. [35] is a mean field approximation of each of the populations of our detailed model (5 x 2800 dimensional). However, in both models the network dynamics is qualitatively the same. This allows us to show that the number of neurons per node does not influence the results at the network level. However, adding a new population, as this implies the appearance of a new loop, could generate new behaviors such as cross-frequency coupling (synchronous interaction between oscillatory activities) due to the interaction between different time scales.

We have not analyzed the dynamical behavior of the system composed by direct and indirect loops. It is certainly possible that some of the details of the results could differ from the ones obtained in our direct-hyperdirect model but we should note that in both cases the oscillations arise from the competition between one globally excitatory loop (the direct one) and one inhibitory (the indirect or the hyperdirect) [44, 69–71]. From this point of view both cases can be equally represented by our reduced two-population model. It is worth noting that by incorporating biophysical details of the BG network could lead to predictions on the optimal stimulation pattern more specific than those obtained with our system-level approach. Among the additional aspects that can be studied are the intra-area connectivity patterns that have been ignored in this work. We have not considered those connections here because we have focused on the system level architecture, that is certainly dominated by inter-area connections. However, detailed intra-area connectivity patterns could be relevant for aspects such as action selection [72, 73], that we have not analyzed here. Another simplification is the generation of the parkinsonian state via the depletion of 90% in the level of dopamine, which affects the strength of only one set of synaptic efficacies and one set of thresholds. Here we are taking a minimalistic approach given that this approximation is enough to generate exaggerated oscillations in the *β*-band and loss of action selection in the spatially structured model which is an extended version of the pathophysiology model of PD previously reported in [35].

The analytical and numerical results presented in this work indicate that there are several possible mechanisms for suppression of exaggerated oscillations in the *β*-band. One of them is zero-pole cancellation which, though mathematically possible, has little relevance as a common key DBS mechanism at the system-level. We found that for clinically relevant DBS parameters (*f*_*DBS*_ > 100 Hz, *δ* ∼ 60-100 *μ*s), the pole-zero cancellation produces the suppression of frequencies not in the *β*-band but in the *γ*-band. Besides, the linear state condition for the neural populations required by the pole-zero cancellation mechanism can hardly be reconciled with the empirical concept stating that HFS produces a functional inhibition of the target nucleus, as its effects mimic what is functionally obtained with the ablative lesion or blockade of the target nucleus [26, 46].

The relevant DBS mechanism we have identified here is a non-linear phenomenon which involves the resetting of the *amplitude* of the oscillations. Each time a stimulation pulse arrives the amplitude of the intrinsic oscillatory activity is suddenly perturbed, and if the frequency of stimulation is high enough, the neural network has no time to re-initiate the oscillation between successive stimulation pulses. This mechanism only works in a well defined range of stimulation frequencies. The stimulation frequency must be high enough to induce resetting but not too high to lead to permanent suppression of activity in some of the populations, leading to a state where the basal ganglia-thalamus-cortex system could not function properly. The proposed mechanism also explains why non-periodic stimulation is less efficient in controlling *β*-oscillations. Keeping the same average stimulation frequency, irregular patterns will have longer intervals without stimulating pulses than regular ones, leading to more time for the oscillations to recover. This is also supported by the results obtained increasing the mean stimulation frequency of the aperiodic stimulation pattern while keeping the degree of irregularity constant (data not shown). In this case, the efficacy of the aperiodic stimulation pattern to suppress the pathological *β*-band oscillations is increased as the mean stimulation frequency is augmented since the occurring number of inter-pulse intervals that are long enough for the system to recover the oscillatory dynamics is diminished.

From the theoretical analysis presented in this work we obtain not only the observed range of stimulation frequency that has clinical relevance but also several predictions that can be tested to evaluate the validity of our approach:

- Emergence of the resetting of the amplitude of the oscillations as a system-level mechanism: The results presented in this work in relation to the proposed resetting mechanism are emergent properties of the BG anatomy at the system-level that can be addressed without taking into account biophysical details of the relevant structures. Thus, the resetting of the *β*-oscillations elicited by the HFS emerges as a global effect in the interconnected structure of the BG-thalamocortical network. The latter is consistent with the evidence suggesting that HFS affects multiple structures simultaneously [53].
- Relative parameter values that give rise to suppression of *β*-oscillations: The results regarding the resetting mechanism show that for a stimulation with parameters $H_{0}^{DBS}$, *f*_*DBS*_, *δ* the system can be in one of the following three states: Inactive network, Active network with oscillations suppressed, Active network with oscillations. In particular, it is possible to identify for which values of the stimulation parameters the oscillations are suppressed without nullify the activity. We found that our model successfully reproduces the observed clinically relevant range for the frequency of the stimulation (30 Hz < *f*_*DBS*_ < 220 Hz) and pulse width (*δ* << 1/*f*_*DBS*_). According to the results obtained, the network will be active as long as the power of the stimulation signal is less than a threshold value. Otherwise, the network is inactive and dynamic does not change, independently of the particular values of the parameters.
- Period doubling dynamics for an appropriate range of stimulation frequency: The clinical evidence show that the PD symptom improvement is typically achieved using stimulation frequencies higher than 100 Hz. In most cases, other parameter configurations have no effect or aggravate the symptoms. It would be clearly interesting to experimentally explore if stimulation frequencies *f*_*DBS*_ below *f*_*d*_ are linked to the increase of *β*-oscillations via a period doubling bifurcation elicited by the interaction between the *β*-oscillations and the periodic stimulation pattern.
- Difference in the stimulation frequency that generates suppression of activity when DBS is inhibitory: Theoretical and experimental evidence have shown that electrical DBS may perform both excitation and inhibition on different compartments of the same neurons (soma, dendrite, or axon) or on different neurons depending on their orientations relative to the electrical field [50, 74]. This motivates us to explore the response of our models under inhibitory stimulation patterns. In the case of the detailed network model, we simulated excitatory and inhibitory periodic stimulation patterns applied to the STN nuclei. In the case of the reduced model simulations were performed for the four combinations corresponding to excitatory/inhibitory stimulation applied in the neuronal population *N*_1_/*N*_2_. For both computational models (detailed and reduced), it was found that inhibitory ($H_{0}^{DBS}<0$) and periodic stimulation patterns produce a similar activity resetting mechanism, however, the stimulation frequency of interest, i.e. suppressed *β*-Power and non-null activity, is significantly higher *f*_*DBS*_ > 500 Hz (data not shown) than that observed in the excitatory case *f*_*DBS*_ > 30 Hz (Figs 4 and 9).
- Effect of the pulse shape: Although the study focused on two types of monopolar pulses, square and triangular, it is expected that in the very small width limit, all pulses have the same effect (regardless of their shape). This implies that the defined states would not be modified. Moreover, for pulses of finite width the criterion mentioned above regarding the stimulation power threshold value for the activity suppression remains valid.

The mechanism of resetting of amplitude of the oscillations can be contrasted with those involving resetting of the *phase* of the oscillations in connection with open-loop [75] and closed-loop [52, 76–78] DBS paradigms. For instance, the phase resetting approaches of [78] involves ensemble of coupled Kuramoto phase-oscillators [79] and includes linear and non-linear feedback. A related approach regarding the open-loop DBS mechanism is considered in [75], where the emphasis is put on the desynchronizing effects of the stimulation due to the interaction with the intrinsic oscillatory mechanism of globus pallidus neurons. In the target nuclei neurons are not interacting and the stimulation tends to cancel the synchronizing effect of a common input. It would be clearly interesting to implement this approach in the context of a network architecture such as the one we use in this paper.

Let us emphasize that in this work we have focused on exaggerated *β*-oscillations as proxy for parkinsonian pathology. Extensive evidence indeed exists indicating that abnormally synchronized activity patterns in the *β*-band (13-35 Hz in humans) are a hallmark of untreated PD [16–19]. Besides, there is also evidence showing that in a significant number of patients/cases, HFS allows alleviation of the PD motor symptoms concurrently with the reduction of exaggerated oscillations in the *β*-band [16]. In addition, it is worth noting that the clinical relevance of the HFS, above 100 Hz, seems to be especially puzzling given that the frequency of exaggerated oscillations in the *β*-band is at least three times lower. The latter is a major open issue whose study is relevant for the understanding of the underlying dynamics regarding the stimulated BG nuclei, and this remains true independently from the fact that the functional significance of excessive *β*-oscillations in terms of a mechanistic causal connection with the symptoms of PD has not yet been demonstrated. In fact, our spatially structured model (in which we extend the model previously reported in [35]) predicts that the loss of selection ability occurs before the appearance of oscillations, suggesting that PD motor impairments are not exclusively related to abnormal oscillatory activity in the *β*-band.

The mechanism of resetting of amplitude of the oscillations has implications regarding the mechanism of action of DBS. It supports the hypothesis that therapeutic HFS regularizes neural firing patterns across the cortico-BG-thalamo-cortical circuit overriding the pathological oscillations in the *β*-band and the resulting informational lesion prevents the pathological activity from being transmitted within the BG [51]. Taken together, the results obtained with our reduced and detailed models provide supporting computational and analytical rationale into clinically relevant range for both the frequency and the intensity of the HFS in connection with open-loop DBS paradigms. On the other hand, taking account that the resetting mechanism acts at the system-level, it could be possible to identify the state of the network (oscillatory or non-oscillatory) by processing the LFP signal measured in different BG nuclei. This prediction has implications regarding the development of the envisioned implantable devices capable to operate in a closed-loop manner in order to deliver an adaptive stimulation. In particular, the electrodes (sensing and stimulation) required by the closed-loop device could be placed in the same target nuclei using a single DBS lead. Closed-loop DBS paradigms [75–77, 80–82] could provide more parsimonious adaptive and/or on-demand stimulation patterns capable of minimize the energy delivered to the neural tissue and improve the symptoms by partially restore the pre-pathological state of the BG-thalamocortical network. It is essential to note that, similarly to the state change of the BG network elicited by the current drug-based treatment of PD, the BG-thalamocortical network under open-loop DBS paradigms is not necessarily restored to pre-pathological states. Instead, the network is shifted to some third state of stimulus-synchronized regular pattern of neuronal activity that allows for symptoms to improve relative to the diseased state [51].

## Supporting information

S1 Appendix Volume of tissue activated. (PDF)

S2 Appendix Pole-zero cancellation mechanism. (PDF)

S3 Appendix Frequency for activity suppression. (PDF)

S4 Appendix Frequency for period doubling. (PDF)
