# Supplementary material for: Mechanisms for pattern specificity of deep-brain stimulation in Parkinson’s disease
Source: PLoS One. 2017 Aug 16;12(8):e0182884. doi: 10.1371/journal.pone.0182884 (PMC5558964; doi:10.1371/journal.pone.0182884)
Supplement: S1 Appendix — (PDF) [file pone.0182884.s001.pdf]

Mechanisms for pattern specificity of deep-brain stimulation in Parkinson's disease

Osvaldo Matías Velarde, Germán Mato, Damián Dellavale*.

Centro Atómico Bariloche and Instituto Balseiro, Consejo Nacional de Investigaciones Científicas y Técnicas (CONICET), Comisión Nacional de Energía Atómica (CNEA), 8400 San Carlos de Bariloche, Río Negro, Argentina.

*dellavale@cab.cnea.gov.ar

S1 Appendix. Volume of tissue activated.

Typical values of effective volume for stimulating electrodes v_{ef} are about 100 mm^3 [1]; while for sensing electrodes, $v_{ef} \sim 40$ mm^3 [2]. In our simulations, these values were compared with the real size of the basal ganglia using the rule

$$\frac{vol_{ef}}{vol_{\alpha}} = \frac{\theta_{ef}^{\alpha}}{2\pi}. \quad (1)$$

In the S1 Table, the volumes of each neuron group used in the Eq 1 are indicated.

S1 Table. Volume of different neuron populations.

Ganglio α	vol_{α} (mm^3)
C	36900
STN	195
St	8735
GPi	668
Th	10981

References

1. Schmidt C, Grant P, Lowery M, van Rienen U. Influence of uncertainties in the material properties of brain tissue on the probabilistic volume of tissue activated. IEEE Trans Biomed Eng. 2013;60(5):1378–1387.

2. Logothetis NK. The underpinnings of the BOLD functional magnetic resonance imaging signal. J Neurosci. 2003;23(10):3963–3971.
