# Supplementary material for: Mechanisms for pattern specificity of deep-brain stimulation in Parkinson’s disease
Source: PLoS One. 2017 Aug 16;12(8):e0182884. doi: 10.1371/journal.pone.0182884 (PMC5558964; doi:10.1371/journal.pone.0182884)
Supplement: S2 Appendix — (PDF) [file pone.0182884.s002.pdf]

Mechanisms for pattern specificity of deep-brain stimulation in Parkinson's disease

Osvaldo Matías Velarde, Germán Mato, Damián Dellavale*.

Centro Atómico Bariloche and Instituto Balseiro, Consejo Nacional de Investigaciones Científicas y Técnicas (CONICET), Comisión Nacional de Energía Atómica (CNEA), 8400 San Carlos de Bariloche, Río Negro, Argentina.

*dellavale@cab.cnea.gov.ar

S2 Appendix. Pole-zero cancellation mechanism.

Under conditions $I_i > T_i (i = 1, 2)$, the system is in the linear state (both neurons are active). The differential equations of the reduced model were Laplace transformed to obtain the following simultaneous algebraic equations,

$$\tau_i s \hat{m}_i(s) = -\hat{m}_i + G_j \hat{m}_j(s) e^{-s\Delta_j} + \hat{H}_i(s) - \frac{T_i}{s}. \tag{1}$$

In considering the two neural populations constituting our reduced model, the previous equations can be written in matrix form,

$$\begin{bmatrix} \hat{m}_1 \\ \hat{m}_2 \end{bmatrix} = \frac{1}{p(s)} \begin{bmatrix} 1 + s\mu\tau & G_2 e^{-s\Delta_2} \\ G_1 e^{-s\Delta_1} & 1 + s\tau \end{bmatrix} \begin{bmatrix} \hat{H}_1(s) - \frac{T_1}{s} \\ \hat{H}_2(s) - \frac{T_2}{s} \end{bmatrix}.$$

where s is the Laplace complex variable and $p(s)$ is given by the Eq (3) of the main text.

It was assumed that H_1 is a constant and H_2 is a pulse train with period $\frac{1}{f_{DBS}}$, pulse time δ and amplitude H_0^{DBS}. Therefore, their Laplace transforms are

$$\begin{aligned} \hat{H}_1(s) &= \frac{H_1}{s}, \\ \hat{H}_2(s) &= \frac{H_0^{DBS}}{s} \frac{1 - e^{-s\delta}}{1 - e^{-s/f_{DBS}}}. \end{aligned} \tag{2}$$

Also, we assumed that system have an imaginary pole $i\omega$, i.e. there is an imaginary root of $p(s)$.

Solving the equation $\hat{m}_1(i\omega) = 0$ in ω yields,

$$\hat{H}_2(i\omega) = -\frac{1 + i\omega\mu\tau}{G_2} e^{i\omega\Delta_2} \left(\hat{H}_1(i\omega) - \frac{T_1}{i\omega} \right) + \frac{T_2}{i\omega} \tag{3}$$

Then, using Eq (2), we find that

$$H_0^{DBS} \frac{\sin(\omega\delta/2)}{\sin(\omega/2f_{DBS})} e^{i\omega(\frac{1}{f_{DBS}} - \delta)/2} = -\frac{1 + i\omega\mu\tau}{G_2} e^{i\omega\Delta_2} (H_1 - T_1) + T_2 = z = |z| e^{i\phi(z)} \tag{4}$$

where z is a complex number that depends only on the parameters of the network.
