# Supplementary material for: Mechanisms for pattern specificity of deep-brain stimulation in Parkinson’s disease
Source: PLoS One. 2017 Aug 16;12(8):e0182884. doi: 10.1371/journal.pone.0182884 (PMC5558964; doi:10.1371/journal.pone.0182884)
Supplement: S3 Appendix — (PDF) [file pone.0182884.s003.pdf]

Mechanisms for pattern specificity of deep-brain stimulation in Parkinson's disease

Osvaldo Matías Velarde, Germán Mato, Damián Dellavale*.

Centro Atómico Bariloche and Instituto Balseiro, Consejo Nacional de Investigaciones Científicas y Técnicas (CONICET), Comisión Nacional de Energía Atómica (CNEA), 8400 San Carlos de Bariloche, Río Negro, Argentina.

*dellavale@cab.cnea.gov.ar

S3 Appendix. Frequency for activity suppression.

First, we suppose that H_2 is such that $I_1 < T_1$ at all time. Under this condition, the steady state of the system is given by $m_1 \to 0$ in Eq (1) of the main text. On the other hand, the equation for m_2 (see Eq (2) in the main text) is reduced to,

$$\tau_2 \dot{m}_2 = -m_2 + H_2(t) - T_2.$$

Then,

$$m_2(t) = e^{-\frac{t}{\tau_2}}(m_2(0) + \frac{1}{\tau_2}\int_0^t H_2(s) e^{\frac{s}{\tau_2}} ds - T_2(e^{\frac{t}{\tau_2}} - 1)),$$

where $m_2(0)$ is a constant of integration.

Taking into account the periodicity in time, we can to determine the constant $m_2(0)$ given by the following expression

$$m_2(0) = H_0^{DBS} \frac{e^{\frac{\delta}{\tau_2}} - 1}{e^{\frac{1}{f_{DBS}\tau_2}} - 1} - T_2.$$

Since the input I_1 is lower than threshold T_1, it follows that $I_1 = G_2 m_2(0) + H_1 < T_1$. This condition is equivalent to determine a lower bound for the stimulation frequency f_{DBS}

$$f_{DBS} > \frac{1}{\tau_2 \lg(1 + H_0^{DBS} \frac{G_2}{T_2 G_2 + T_1 - H_1}(e^{\frac{\delta}{\tau_2}} - 1))}.$$

In the limit of δ sufficiently small,

$$f_{DBS} > f_s = \frac{1}{\tau_2 \lg(1 + \frac{H_0^{DBS}\delta}{\tau_2} k)} \sim \frac{1}{H_0^{DBS}\delta k}.$$

where $k = \frac{G_2}{T_2 G_2 + T_1 - H_1}$.
