# Supplementary material for: Mechanisms for pattern specificity of deep-brain stimulation in Parkinson’s disease
Source: PLoS One. 2017 Aug 16;12(8):e0182884. doi: 10.1371/journal.pone.0182884 (PMC5558964; doi:10.1371/journal.pone.0182884)
Supplement: S4 Appendix — (PDF) [file pone.0182884.s004.pdf]

Mechanisms for pattern specificity of deep-brain stimulation in Parkinson's disease

Osvaldo Matías Velarde, Germán Mato, Damián Dellavale*.

Centro Atómico Bariloche and Instituto Balseiro, Consejo Nacional de Investigaciones Científicas y Técnicas (CONICET), Comisión Nacional de Energía Atómica (CNEA), 8400 San Carlos de Bariloche, Río Negro, Argentina.

*dellavale@cab.cnea.gov.ar

S4 Appendix. Frequency for period doubling.

In considering the reduced model in the linear state, the Eqs (1) and (2) of the main text can be written as follows,

$$\tau_i \dot{m_i} = -m_i + G_j m_j(t - \Delta_j) + H_i(t) - T_i$$

where $i \neq j$ and $i = 1, 2$.

We perform a Fourier expansion for solution m_i and external inputs H_i. We consider that H_i is a T-periodic function and m is a 2T-periodic function. Then,

$$H_i(t) = \sum_{n\in\mathbb{Z}} b_{i,n} \exp(\frac{2\pi n}{T}\mathbf{i}t),$$
$$m_i(t) = \sum_{n\in\mathbb{Z}} a_{i,n} \exp(\frac{\pi n}{T}\mathbf{i}t).$$

Thus, in the frequency domain we obtain the following linear equation system,

$$\sum_{n\in\mathbb{Z}} \exp(\frac{\pi n}{T}\mathbf{i}t)\left[a_{i,n}(1+\tau_i\frac{\pi n}{T}\mathbf{i}) - G_j a_{j,n}\exp(-\frac{\pi n}{T}\mathbf{i}\Delta_j)\right] = \sum_{n\in\mathbb{Z}} \exp(\frac{2\pi n}{T}\mathbf{i}t) b_{i,n} - T_i.$$

By comparing terms corresponding to the same frequency we obtain,

$$a_{i,n}(1+\tau_i\frac{\pi n}{T}\mathbf{i}) - G_j a_{j,n}\exp(-\frac{\pi n}{T}\mathbf{i}\Delta_j) = \begin{cases} b_{i,0} - T_i & n = 0 \\ 0 & n \text{ odd number} \\ b_{i,n/2} & n \text{ even number} \end{cases}$$

In particular, m_i will be 2T-periodic, if $a_{i,\pm1} \neq 0$. Then, it follows that,

$$\begin{bmatrix} (1 \pm \tau_1 \frac{\pi}{T}\mathbf{i}) & -G_2 \exp(\mp\frac{\pi}{T}\mathbf{i}\Delta_2) \\ -G_1\exp(\mp\frac{\pi}{T}\mathbf{i}\Delta_1) & (1 \pm \tau_2\frac{\pi}{T}\mathbf{i}) \end{bmatrix} \begin{bmatrix} a_{1,\pm1} \\ a_{2,\pm1} \end{bmatrix} = \begin{bmatrix} 0 \\ 0 \end{bmatrix}.$$

The previous equation have an infinite number of solutions, that is, the determinant of the right hand side matrix is zero. As a consequence we obtain,

$$p(s = \frac{\pi}{T}\mathbf{i}) = 0 \Rightarrow \frac{\pi}{T} = 2\pi(\frac{\omega}{2\pi}) \Rightarrow f_d = 2(\frac{\omega}{2\pi})$$
